# Enhancing mass spectrometry imaging accessibility using convolutional autoencoders for deriving hypoxia-associated peptides from tumors

**DOI:** 10.1038/s41540-024-00385-x

**Published:** 2024-05-27

**Authors:** Verena Bitto, Pia Hönscheid, María José Besso, Christian Sperling, Ina Kurth, Michael Baumann, Benedikt Brors

**Affiliations:** 1 Division of Applied Bioinformatics, German Cancer Research Center (DKFZ), Heidelberg, Germany; 2 Division of Radiooncology/Radiobiology, German Cancer Research Center (DKFZ), Heidelberg, Germany; 3 HIDSS4Health – Helmholtz Information and Data Science School for Health, Karlsruhe/Heidelberg, Heidelberg, Germany; 4 Faculty for Mathematics and Computer Science, Heidelberg University, Heidelberg, Germany; 5 National Center for Tumor Diseases (NCT), Partner Site Dresden, German Cancer Research Center (DKFZ), Heidelberg, Germany; 6 University Hospital Carl Gustav Carus (UKD), Technische Universität Dresden, Institute of Pathology, Dresden, Germany; 7 Faculty of Medicine and University Hospital Carl Gustav Carus, Technische Universität Dresden, Dresden, Germany; 8 OncoRay – National Center for Radiation Research in Oncology, Faculty of Medicine and University Hospital Carl Gustav Carus, Technische Universität Dresden, Helmholtz-Zentrum Dresden - Rossendorf, Dresden, Germany; 9 German Cancer Consortium (DKTK), Core Center Heidelberg, Heidelberg, Germany; 10 National Center for Tumor Diseases (NCT), Heidelberg, Germany; 11 Medical Faculty Heidelberg and Faculty of Biosciences, Heidelberg University, Heidelberg, Germany

**Keywords:** Cancer, Computational biology and bioinformatics

## Abstract

Mass spectrometry imaging (MSI) allows to study cancer’s intratumoral heterogeneity through spatially-resolved peptides, metabolites and lipids. Yet, in biomedical research MSI is rarely used for biomarker discovery. Besides its high dimensionality and multicollinearity, mass spectrometry (MS) technologies typically output mass-to-charge ratio values but not the biochemical compounds of interest. Our framework makes particularly low-abundant signals in MSI more accessible. We utilized convolutional autoencoders to aggregate features associated with tumor hypoxia, a parameter with significant spatial heterogeneity, in cancer xenograft models. We highlight that MSI captures these low-abundant signals and that autoencoders can preserve them in their latent space. The relevance of individual hyperparameters is demonstrated through ablation experiments, and the contribution from original features to latent features is unraveled. Complementing MSI with tandem MS from the same tumor model, multiple hypoxia-associated peptide candidates were derived. Compared to random forests alone, our autoencoder approach yielded more biologically relevant insights for biomarker discovery.

## Introduction

Spatial omics emerges as a promising tool to characterize intratumoral heterogeneity in solid cancers. For example, mass spectrometry imaging (MSI) allows measuring spatially resolved peptides, metabolites or lipids directly from tissue^1^. With this technology, molecules in a samples are ionized, which allows to separate the ions based on their mass-to-charge ratio (m/z) by means of a mass analyzer. Depending on the mass analyzer used, e.g., time of flight (TOF) mass spectrometry (MS) or Fourier transform ion cyclotron resonance (FT-ICR) MS, different mass resolving power, mass accuracy, and mass range can be achieved^2^. In case of TOF-based MS, the mass accuracy and mass resolving power are too weak to directly identify molecules like peptides. To overcome this limitation, m/z values of MSI are therefore often complemented by tandem mass spectrometry (MS) experiments^3^. Compared to other spatial omics technologies, MSI is still rarely used for biomarker discovery, likely because m/z values are difficult to interpret. Additionally, the spatial resolution has increased considerably in the last decades, imposing new challenges on analyses^4^. From a computational perspective, spatial omics data represents a high-dimensional, highly correlated feature space. Often, these experiments are carried out on one or only few samples, making (spatial) omics data a representative of what is known as “small n, large p” problems. Traditional statistical models, like linear regression, were designed for opposing problems (”large n, small p”) and thus are not applicable to omics data^5^.

One common approach to analyze omics data is first reducing the dimensionality of data by dismissing uninformative features. This is carried out either by selecting or extracting features.

With feature selection methods, the most promising original features are chosen and remain intact. The most basic methods, including univariate filtering, ignore feature dependencies^6^ such as co-location of ions in the case of MSI. Other techniques incorporate also collective information. For example, random forest (RF) models make predictions based on the ensemble of all trees in a forest, enabling to capture non-linear relationships between features. In practice, supervised feature selections methods, in which class labels are incorporated into the selection process, are designed to identify a subset of discriminative features, but not every important feature^7^. This is particular the case for correlated features as their precise contributions to a statistical model are difficult to derive^8^. In tree-based models, even in the obvious case of replicates, this leads to differences in their assigned feature importance^7^, hampering interpretability and reproducibility of results. Moreover, in the case of MSI, one feature may represent only partial information about a biological trait. For example, enzymatic digestion is a frequently applied method for sample preparation to increase the resolution power when measuring proteins^3^. As a result, one intact peptide is split into multiple tryptic peptides, i.e., multiple m/z values. Identifying only a small number of m/z values as relevant in a supervised task restricts the ability to infer the actual peptide of interest.

Feature extraction methods, e.g., unsupervised clustering, principle component analysis (PCA) or autoencoders, follow a different approach by aggregating multiple features to create new representatives. In case of autoencoders, this is accomplished by compressing the input data into a lower dimensional space, the so-called latent space, by means of an encoder and a decoder. While feature extraction can capture multicollinearity well, the results are often difficult to interpret and lack target-specific information. Also, unsupervised extraction procedures may not necessarily aggregate the features of interest^9^ if those are not expressed predominantly. For example, it was previously shown that PCA or unsupervised clustering applied on MSI might be helpful to complement histopathology by revealing distinct tissue morphologies or heterogeneity^10,11^. However, this also suggests that less pronounced features are likely to be missed.

In this paper, we investigated if MSI allows to detect signals of tumor hypoxia and whether autoencoders retain this information in a lower dimensional space. Tumor hypoxia is a state of low oxygen levels in solid tumors that is associated with poor prognosis^12^. As hypoxia can arise in all kind of cells, in different tumor regions, and at varying degree, it is likely that other structural features in tissue, such as tissue morphology, cause more pronounced signals in MSI. The data studied represent tryptic peptide information from untreated tumors of one head and neck squamous cell carcinoma (HNSCC) xenograft model. We showed that, depending on the hyperparameters chosen, autoencoders produce more valuable insights in tumor hypoxia compared to RFs alone. The use of autoencoders also minimized aggressive pre-processing and thereby retained a comprehensive view of the MSI data. In combination with the outlined recovery method to link original features to a latent feature of interest, this facilitated detecting peptide candidates from complementing tandem MS data.

## Results

**Fig. 1:**
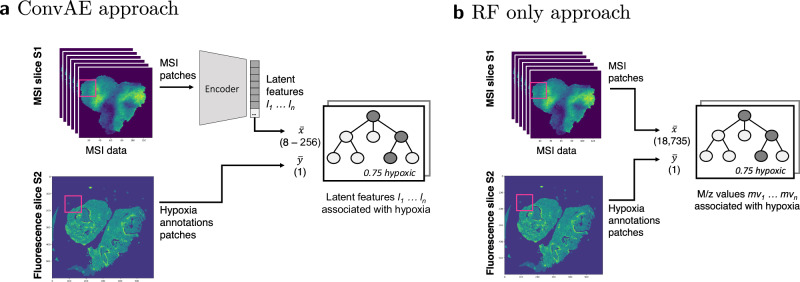
Workflow to identify hypoxia-associated peptides from mass spectrometry imaging (MSI) experiments. Numbers denote the length of the vector, i.e., 8–256 latent space features or 18,735 original features. **a** Convolutional autoencoder (ConvAE) approach: MSI data of multiple samples were encoded with the previously trained ConvAE. Random forest (RF) regression models were trained on the encoded data and the hypoxia annotations from consecutive slices by taking the mean of 2 × 2 data pixels and 3 × 3 annotation pixels respectively. **b** RF only approach: Patches of MSI data and hypoxia annotations were used to train a RF regression model by taking the mean of 3 × 3 data and annotations pixels.

**Fig. 2:**
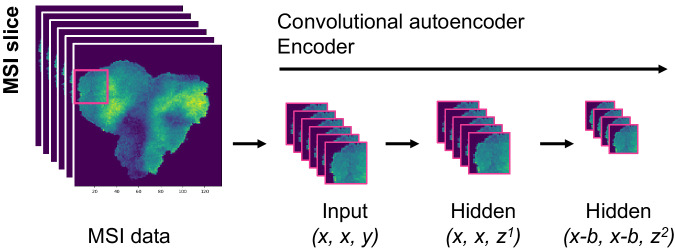
Encoding of mass spectrometry imaging (MSI) data. Data was cut into patches of size 3 × 3 pixels. The autoencoder was trained on overlapping patches (step size of 2) using the following configuration: x = 3, y = 18,735, z^1^ = 1024, b = 1, z^2^ = 8–256. The original 18,735 mass-to-charge (m/z) values are thereby reduced with a first hidden convolutional layer to 1024. The second hidden convolutional layer reduces the patches from 3 × 3 pixels to 2 × 2 pixels and the feature space from 1024 to 8–256.

We analyzed if features associated with hypoxia are recognizable in MSI experiments from tissue of five HNSCC xenograft samples. For every sample, one slice underwent MSI and a consecutive slice was stained with pimonidazole as a biochemical marker of hypoxic cells. Hypoxic regions were segmented and co-registered to the MSI data. MSI data was pre-processed and a total of 18,735 peaks per pixel were retained. With the aim of identifying features associated to hypoxia, we compared a convolutional autoencoder (ConvAE) to a random forest (RF) only approach (Fig. 1). In both cases, random forest regression models were trained to assess the feature importance (FI) for predicting hypoxia. In the ConvAE approach, an autoencoder was first trained on MSI patches (3 × 3 pixels) to learn lower dimensional representations of m/z values (2 × 2 pixels, Fig. 2). Next, sampled hypoxic and non-hypoxic MSI patches were encoded using the trained ConvAE. The encoded MSI patches and their corresponding hypoxia annotation patches were inputted into the regression model. For the RF only approach, feature encoding was disabled. For a compatible input size, each MSI patch was reduced to its mean per m/z value. The processing of the annotations patches remained unchanged.

First, we compare qualitative results from one individual ConvAE to one RF only run. Complementing, we assess quantitative metrics across 10 runs.

## Qualitative results

### ConvAE: Hypoxia-associated latent feature

After training the ConvAE with a latent space size of 64, latent feature #56 exhibited the highest FI for hypoxia. Supplementary Fig. 1 shows a visual representation of the hypoxia-associated latent feature (left) and one latent feature with moderate association for comparison (right).

**Fig. 3:**
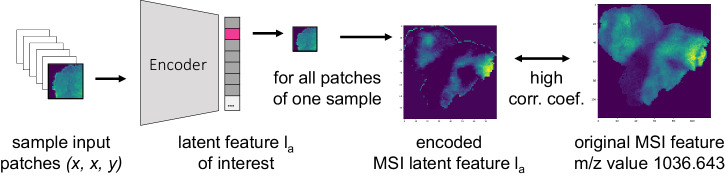
Recovery of feature information. For each sample, its modified patches are encoded and its latent space representations compared to the original features using the Spearman correlation coefficient.

**Fig. 4:**
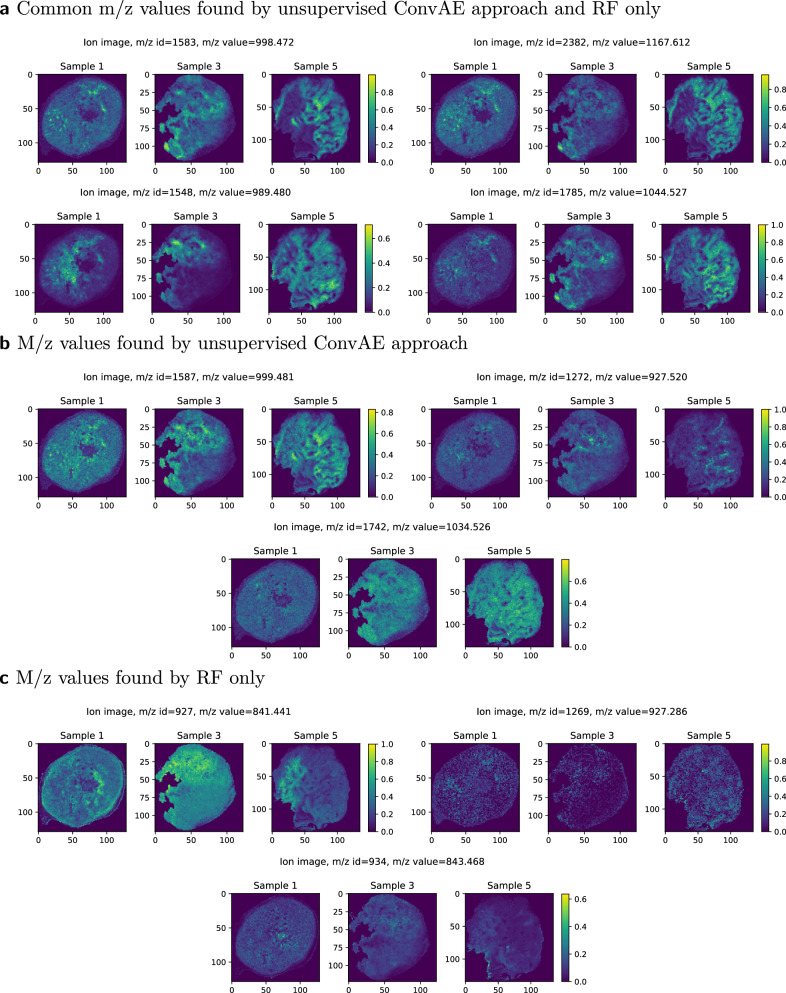
Exemplary mass-to-charge (m/z) values associated with hypoxia. Features that, (**a**) were found by the unsupervised convolutional autoencoder (ConvAE) and the random forest (RF) only approach, (**b**) were distinctively found by the unsupervised ConvAE approach, (**c**) were distinctively found by the RF only approach.

**Fig. 5:**
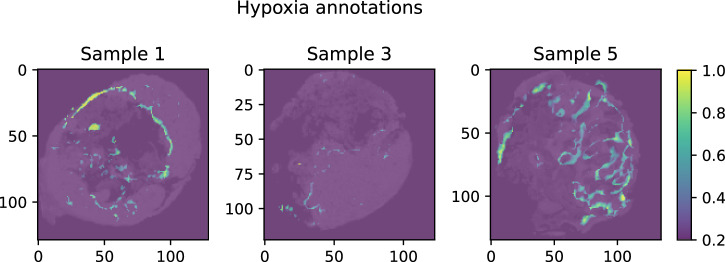
Hypoxia annotations of individual samples. Yellow = high degree of hypoxia, dark blue = no hypoxia.

A follow-up recovery method was implemented (Fig. 3) to identify m/z values that contributed to latent feature #56. For all patches of a given sample, one m/z value remained unchanged while the intensities of all other m/z values for all pixels were set to 1. The modified patches were encoded accordingly. The encoded image of latent feature #56 was compared against the original ion image using the Spearman correlation coefficient. M/z values linked to a latent feature were expected to show high correlation coefficients, while unrelated ones were expected to have low correlations. The procedure was repeated for all m/z values. We defined associations with the latent feature using a cutoff value of >0.95 to reduce noisy associations. A total of 180 m/z values were found to contribute to the hypoxia-associated latent feature #56. Some exemplary m/z values are shown in Fig. 4a, b, with the corresponding hypoxia annotations in Fig. 5.

### RF only: Hypoxia-associated m/z values

We disabled the autoencoder feature encoding and extracted the FI from the regression model accordingly. M/z value 998.472 achieved the highest FI (in 4 out of 10 cross validation runs). For comparison with the ConvAE approach, we defined a cutoff based on the highest ranked feature score to retrieve a comparable amount of m/z values. We considered all m/z values of high importance which reached at least one fourth of the score in one cross validation run. This resulted in a total of 156 m/z values considering all cross validation runs.

### Comparison of hypoxia-associated m/z values

**Fig. 6:**
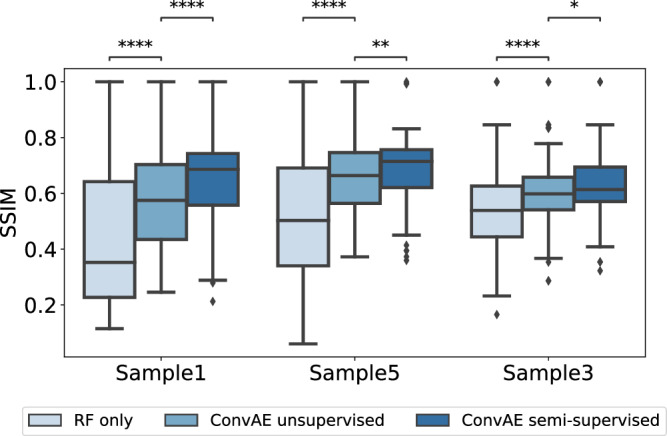
Qualitative analysis of exemplary runs of convolutional autoencoder (ConvAE) and random forest (RF) only approaches. Boxplots show the distribution of the structural similarity index measure (SSIM) of all identified hypoxia-associated features (156 in RF only versus 180 in unsupervised ConvAE approach versus 120 in the semi-supervised ConvAE approach) to the reference mass-to-charge (m/z) value 998.472 per sample. Boxplots follow the Tukey style (see Methods), incorporating *p* value cutpoints: **** *<* 10^−4^, *** *<* 0.001, ** *<* 0.01, * *<* 0.05, ns *>*= 0.05. Groups were compared using two-sided Mann–Whitney U rank tests, where *p* values were corrected to control the false discovery rate.

From the 156 m/z values associated with hypoxia in the RF only approach, 53 of the m/z values were also identified in the ConvAE approach (Fig. 4a). As expected, peaks which we consider as replicate m/z values due to mass shifts (see Methods) were not obtaining an identical score in the RF only approach, but were found within the defined cutoff (e.g., m/z values 998.472 and 998.502). More critically, isotopes (e.g., m/z values 999.481, an isotope of m/z value 998.472) were not retained in contrast to the ConvAE approach (compare Fig. 4b). Other m/z values received a high score without showing clear associations to the actual annotations (Fig. 4c, compare Fig. 5) in the RF only approach. To compare all associated m/z values systematically, the structural similarity index measure (SSIM) for each m/z value associated with hypoxia against the highest ranked RF m/z value 998.472 was calculated. A high SSIM score would indicate that the associated features share similar characteristics, which is expected in case all are linked to the hypoxia annotations. Figure 6 highlights that the 180 features identified by the ConvAE approach (denoted as ConvAE unsupervised) exhibited in all samples a significant higher SSIM score than the corresponding features of the RF only approach. Given that the FI metric identifies discriminate features but not necessarily positively correlating features to the hypoxia annotations (compare Fig. 4c, m/z value 841.441), the lower SSIM of the RF only approach is to some extend expected. Thus, compared to the RF only approach, the extraction of features using a convolutional autoencoder discovered a more reliable set of hypoxia-associated m/z values.

### Derived peptide candidates from tandem MS

Complementing MSI experiments, tandem MS was performed to identify possible peptide candidates (see Methods) by mapping MSI masses to tandem MS masses. The quality of this step depends on the number and soundness of MSI m/z values which were associated with hypoxia. From the 180 m/z values associated with hypoxia in the ConvAE approach, 50 peptide candidates were identified where at least two individual MSI masses could be matched to masses of the tandem MS experiment (Table 1). Among these candidates, several have been associated with tumor hypoxia before. For example, phosphoglycerate kinase 1 (PGK1), pyruvate kinase M (PKM), and lactate dehydrogenase A (LDHA), are known to be stimulated by HIF-1_*α*_, a transcription factor involved in adopting to changes in oxygen supply^13^. Genes involved in glucose metabolism were used as surrogate markers in different gene signatures to prognosticate tumor hypoxia, e.g., *ALDOA* is part of the HNSCC hypoxia gene signature of refs. ^14,15^. For many more peptide candidates some connections to hypoxia-induced pathways were found. Others, while not directly linked to tumor hypoxia, have been found more generally associated with poor prognosis. For example, *KRT6A, KRT6B, KRT6C* were used as part of a metagene signature to identify HNSCC patients at high risk for loco–regional recurrences after surgery^16^. Taken together, we showed that our proposed workflow extracts relevant peptides for tumor hypoxia.

**Table 1:** Peptide candidates (#50) found with at least 2 masses matched from unsupervised convolutional autoencoder (ConvAE) run to tandem mass spectrometry experiment, showing only one exemplary mass pair, the complete data is provided as Supplementary Table in a separate file

Protein(s)	Gene name(s)	Mass 1	Mass 2
DNA-dependent protein kinase catalytic subunit	PRKDC	877.466	1337.665
Keratin, type II cytoskeletal 6 A;Keratin, type …	KRT6A;KRT6C;KRT6B	877.441	808.387
Annexin A1	ANXA1	808.400	1063.564
Cytochrome b-c1 complex subunit 1, mitochondrial	UQCRC1	808.400	1042.519
Phosphoglycerate kinase 1	PGK1	808.400	1011.519
Elongation factor 1-gamma	EEF1G	809.402	937.455
Keratin, type II cytoskeletal 5	KRT5	809.402	1409.733
Cullin-associated NEDD8-dissociated protein 1	CAND1	988.480	965.469
Serine hydroxymethyltransferase, mitochondrial;…	SHMT2	988.480	854.495
Heat shock cognate 71 kDa protein	HSPA8	988.480	1409.694
RNA-binding protein with serine-rich domain 1	RNPS1	989.471	864.406
Eukaryotic translation initiation factor 3 subu…	EIF3L	989.471	964.486
Collagen alpha-3(VI) chain	COL6A3	989.471	1036.529
Desmoplakin	DSP	1011.490	944.515
Heterogeneous nuclear ribonucleoprotein R;Heter…	HNRNPR;SYNCRIP	1011.490	926.481
Fatty acid-binding protein, epidermal	FABP5	1042.547	926.520
Prelamin-A/C;Lamin-A/C	LMNA	1042.547	1027.527
Tropomyosin alpha-4 chain	TPM4	1042.547	1259.603
Trifunctional purine biosynthetic protein adeno…	GART	1042.547	1036.548
Eukaryotic translation initiation factor 4 gamma 1	EIF4G1	1026.520	1410.739
L-lactate dehydrogenase A chain	LDHA	1026.520	1166.637
Programmed cell death protein 6	PDCD6	997.502	1338.656
60S ribosomal protein L18a	RPL18A	1042.519	926.520
Eukaryotic translation initiation factor 3 subu…	EIF3C;EIF3CL	1042.519	1166.637
Keratin, type I cytoskeletal 14	KRT14	1036.529	1166.637
Fructose-bisphosphate aldolase A;Fructose-bisph…	ALDOA	1043.548	939.462
60S ribosomal protein L15;Ribosomal protein L15	RPL15	1166.612	880.449
ATP-dependent RNA helicase A	DHX9	990.459	1074.523
Tubulin alpha-1B chain;Tubulin alpha-4A chain;T…	TUBA1B;TUBA1C;TUBA1A;…	1409.733	774.394
Eukaryotic translation initiation factor 3 subu…	EIF3A	1409.733	816.437
Elongation factor 2	EEF2	1012.508	879.436
Pyruvate kinase PKM;Pyruvate kinase	PKM	883.451	1167.621
Heterogeneous nuclear ribonucleoproteins A2/B1	HNRPA2B1;HNRNPA2B1	1409.694	1337.665
Aconitate hydratase, mitochondrial	ACO2	1411.719	921.446
Isoleucine–tRNA ligase, cytoplasmic	IARS	1411.719	957.533
T-complex protein 1 subunit theta	CCT8	998.516	1167.580
Bifunctional glutamate/proline–tRNA ligase;Glu…	EPRS	965.469	1063.564
Keratin, type I cytoskeletal 16	KRT16	1337.665	854.495
EH domain-containing protein 4	EHD4	1337.665	937.455
Keratin, type II cytoskeletal 75	KRT75	921.446	1038.511
Myeloperoxidase;Myeloperoxidase;89 kDa myeloper…	MPO	921.446	937.455
Transketolase	TKT	921.446	944.515
60 kDa heat shock protein, mitochondrial	HSPD1	854.495	1007.493
Annexin A4;Annexin	ANXA4	856.472	1074.523
Actin, cytoplasmic 1;Actin, cytoplasmic 1, N-te…	ACTB;ACTG2;ACTA2;…	944.515	1197.680
26S proteasome non-ATPase regulatory subunit 3	PSMD3	1411.683	957.533
Cyclin-dependent kinase 1	CDK1;CDC2	772.417	1027.527
Transitional endoplasmic reticulum ATPase	VCP	1074.523	1050.523
Heat shock protein beta-1	HSPB1	1074.523	940.463
Activated RNA polymerase II transcriptional coa…	SUB1	1197.680	1259.603

### Specificity of latent features

To assess feature specificity, the proposed recovery method was applied to additional latent features of the ConvAE. Therefore, the latent feature with the second highest FI score for hypoxia (#37), two features with moderate hypoxia association (#26, #44), and one feature with no association (#57) were chosen (see Supplementary Figs. 1, 2). No m/z values were recovered for latent feature #57, separating tissue from background pixels. For all other latent features, Supplementary Fig. 3 shows a comparison based on the SSIM of all recovered m/z values to the reference m/z value 998.472. The figure depicts that latent feature #56 achieved the highest scores, with some high SSIM scores found in all latent features. Latent feature #56 shared 29 recovered m/z values with #37, 20 m/z values with #26 and 15 m/z with #44. The scores of latent feature #56 were significantly higher than those of the second highest latent feature #37. In contrast, latent feature #37 was not significantly different to latent features #26 and #44 in some samples. Inspecting latent feature #37, some negative correlations to the hypoxia annotations can be observed (compare Sample 5), explaining the relatively poor SSIM scores. Hence, for the proposed recovery method, only the top-ranked latent feature was selected to retrieve hypoxia-associated m/z values.

### Semi-supervised ConvAE approach

Given the unsupervised nature of autoencoders, noisy hypoxia associations cannot be entirely ruled out (e.g., m/z value 1034.526, see Fig. 4b). We therefore implemented a semi-supervised approach, in which the error for hypoxic pixels is minimized in addition to the reconstruction error for all pixels (see Methods). We hypothesized that this would force the autoencoder to retain the m/z values associated with hypoxia annotations, rather than focusing on more prominent data characteristics, e.g., tissue morphology. Using our recovery method, 120 m/z values were associated with the hypoxia annotations (Supplementary Fig. 4), from which 75 m/z values were also identified in the unsupervised ConvAE approach. Similar to the RF only approach, we missed some isotope m/z values in the semi-supervised approach. However, comparing the SSIM of all approaches, the hypoxia-associated m/z values of the semi-supervised approach are most similar to the reference m/z value 998.472 (Fig. 6). The lower amount of m/z values also resulted in fewer peptide candidates (Supplementary Table 1). While several hypoxia-associated peptide candidates from the unsupervised run were also present in the semi-supervised approach (like PGK1, LDHA), others were not retained (PKM, ALDOA) and newly ones appeared (GAPDH).

## Quantitative results

### Importance of latent space size

**Fig. 7:**
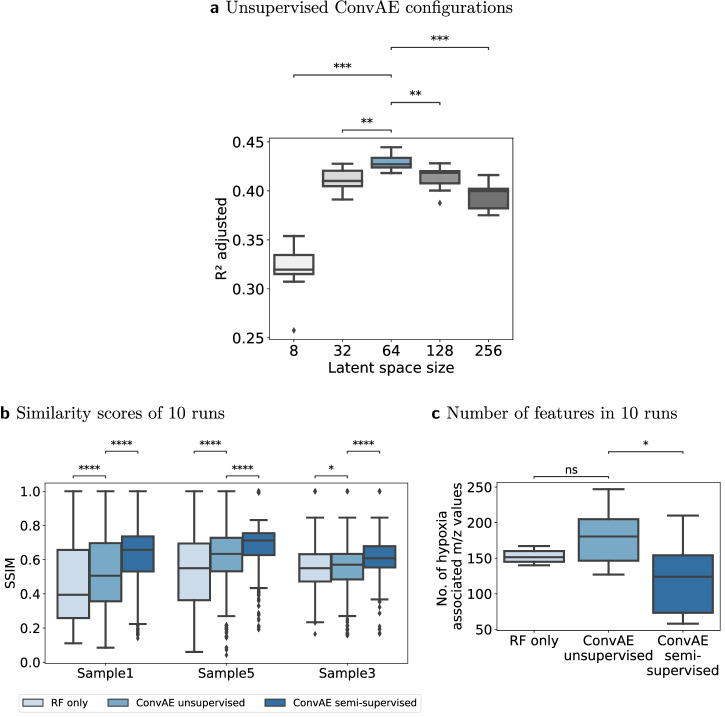
Quantitative analysis of 10 runs each. Boxplots follow the Tukey style (see Methods), incorporating *p* value cutpoints: **** *<* 10^−4^, *** *<* 0.001, ** *<* 0.01, * *<* 0.05, ns >= 0.05. Groups were compared using two-sided Mann–Whitney U rank tests, where *p* values were corrected to control the false discovery rate. **a** Latent space configurations were compared using R^2^ adjusted of the fitted regression models using 10 unsupervised convolutional autoencoder (ConvAE) runs per configuration. **b** Structural similarity index measure (SSIM) of all identified hypoxia-associated features to the reference mass-to-charge (m/z) value 998.472 per sample in 10 individual runs each. **c** Number of hypoxia-associated m/z values that were identified by the three approaches in 10 runs.

The latent space size of the unsupervised ConvAE was chosen to reduce the high-dimensional feature space by leveraging the high multicollinearity among features (see Methods, *Data characteristics*). Starting with 256 latent features, they were steadily decreased to 8. The different configurations were compared in 10 experiments using R^2^ on the trained RF models, adjusted for the number of features in the latent space. A high R^2^ adjusted score would indicate that the fitted models can approximate the hypoxia annotations well. Figure 7a outlines that according to this metric a reasonable latent space size was around 64. Particularly, a low latent space size of 8 failed to capture relevant characteristics to fit a good model for the presented hypoxia annotations. At a latent space size of 128, the variance explained degraded, indicating that no essential further hypoxia-related information can be captured with additional latent features.

### Reproducibility of results

The qualitative results of the ConvAE approaches suggested that they retrieve a more coherent set of hypoxia-related features than RFs alone. However, as some aspects of the training of autoencoders and tree-based models involve randomness, we evaluated how reliable these results can be achieved by comparing 10 runs of the unsupervised ConvAE, semi-supervised ConvAE and RF only approach using the SSIM. Figure 7b shows that the overall findings for the SSIM among approaches can be reproduced. The higher SSIM in the semi-supervised ConvAE approach was achieved by more hypoxia specific latent features, indicated by the lower number of associated m/z values (Fig. 7c). However, it is important to note that we found some ConvAE runs which achieved relatively low SSIM scores for both, the unsupervised and semi-supervised ConvAE approach, although achieving comparable R^2^ scores than other runs. Inspecting those runs showed that top-ranked latent features for hypoxia, while discriminative, were not always positively correlated to the hypoxia annotations or the SSIM reference m/z value. In these cases, other latent m/z values with a slightly lower FI than the top-ranked one showed higher SSIM scores, which can be attributed to the general behavior of tree-based FI metrics.

### Importance of patch size and kernel size

The patch size and kernel size were set such that local structures of hypoxia are emphasized over more global tissue structures. We consider a patch size of 3 × 3 pixels to be the lower bound for a kernel size of 2 × 2 for effectively utilizing the spatial context of surrounding pixels (for details see Supplementary Discussion). Overall, a change in patch size will cause only minor structural changes to the generated latent representations if the kernel size remains fixed (Supplementary Fig. 5 versus Supplementary Figs. 6, 7, Supplementary Table 2). If features of interest are present across larger areas, the patch and kernel sizes should be increased accordingly. However, the regression model’s ability to predict hypoxia will degrade with an increased patch size. This can be attributed to attenuated hypoxia signals and decreased precision in locating them, when mean hypoxia values are derived from a higher total number of pixels in the annotation patches (Supplementary Figs. 8, 9).

### Importance of loss function

In unsupervised dimensionality reduction (DR), low-abundant signals are susceptible to being discarded. The proposed loss function emphasizes to also retain these low intensity signals. To show the relevance of the loss function besides other discussed hyperparameters, a convolutional variational autoencoder (ConvVAE) approach was implemented as alternative. In a variational autoencoder (VAE), the model aims to learn latent representations that approximate a standard Gaussian distribution rather than learning a direct mapping (see Methods). For comparison with the proposed unsupervised ConvAE, the mean vector of the latent feature with the highest FI for hypoxia was chosen in the ConvVAE approach. Supplementary Fig. 10 illustrates that the latent parameters of unsupervised ConvVAEs poorly represent low-abundant signals. This becomes also evident by the significantly lower SSIM scores compared to the non-variational approach across 10 runs (Supplementary Fig. 11).

### Relevance of high feature redundancy

To assess the impact of high feature redundancy on analyses, a stricter pre-processing of data was performed, effectively reducing mass shifts between samples (see Methods). Supplementary Figs. 12, 13 show that even in the case of a reduced set of 2642 and 775 m/z values, the ConvAE approaches achieved higher SSIM scores than without prior DR. In these comparisons, the cutoff for the RF only approaches was set to one-third of the highest score per cross validation run, ensuring the RF only approaches were assigned no more features than the ConvAE approaches (Supplementary Figs. 12b and 13b).

## Discussion

In this work, we highlighted that MSI captures features associated with tumor hypoxia and that convolutional autoencoders can retain these low-abundant features in their latent space by encapsulating highly correlated features. Our presented recovery method tracked back which original m/z values contributed to a latent feature of interest. This was possible by exploiting the shallow ConvAE architecture, which retains the overall spatial structure of MSI data, allowing correlation analysis between original and encoded data. Finally, we complemented the hypoxia-related m/z values with tandem MS data to identify peptide candidates.

Autoencoders have been used to reduce the dimensionality of MSI data previously. However, so far, it was primarily demonstrated that autoencoders can effectively extract predominant signals: Thomas et al. extracted 15 latent features from a mouse brain dataset^17^. Inglese et al. utilized VAE to retrieve 3 latent features from human colon tissue^18^. Likewise, Abdelmoula used VAEs on diverse MSI datasets, like mouse brain models and human prostate cancer tissue, to derive 5 latent features^19^. Matsuda et al. extracted 20 latent features of human corneocytes from TOF secondary ion mass spectrometry (SIMS) imaging with a sparse autoencoder^20^. Similarly, Gardner et al. extracted 20 latent features of a tumor spheroid from TOF-SIMS data through a convolutional autoencoder. All but Gardner et al. utilized a Kullback-Leibler (KL)-divergence term to train the corresponding autoencoder. The KL-divergence, acting as a regularization term, guides the model by prioritizing certain features in the data distribution. Together with a small latent space size as employed in the aforementioned publications, the autoencoder will emphasize more prevalent structures and disregard low-abundant features. This was also apparent in the convolutional VAE presented here, which incorporated KL-divergence and exhibited significantly lower SSIM scores for hypoxia-related features than our proposed ConvAE. Unlike afore-remarked publications, Li. et al. proposed a denoising autoencoder with a large latent space (256 features) and a mean-squared error function to derive features of Listeria species from MS (not MSI)^21^. Similar to other work^22,23^, the extracted features were utilized for classification. However, for biomarker discovery, the explainability of results and the extraction of more than a few discriminative features play a more crucial role than in predictive tasks^24^. We addressed the issue of explainability by providing an intuitive recovery method. Among the discussed publications, only Abdelmoula et al. derived so-called informative m/z peaks to link the latent representation to the original data^19^. Their proposed algorithm is based on a threshold analysis on the weight parameters of the encoder, albeit the quality of the recovery process was not explicitly discussed.

To our knowledge, our work is the first which trains autoencoders on MSI data from multiple samples. This limits the threat of overfitting and allows to learn more robust representations due to the increased sample size. Yet, it introduces challenges like managing sample mass shifts. While heavy pre-processing might reduce the number of potential peaks (see Supplementary Discussion, *Redundancy of m/z values*), it will also introduce artificial masses. Thus, we propose to stick close to the raw measurements, enabling to map the masses of MSI to more precise masses, such as tandem MS.

Combining MSI data with tandem MS data from consecutive tissue slices to link the m/z values to biological compounds was examined in many previous publications. Hoffmann et al. combined MSI data derived from patients with HNSCC with liquid chromatography (LC) MS/MS^25^ to identify markers for malignant cells. They chose 10 characteristic MSI peaks from tumor tissue to correlate them to corresponding peptide candidates. In another targeted approach, metabolites of the hypoxia marker pimonidazole were linked to m/z values of MSI^26^. Other strategies for identifying proteins involve directly recovering them from the used matrix layer by means of tryptic digestion and LC-MS/MS^27^.

Only few publications explored MSI to investigate tumor hypoxia. Djidja et al. combined label-based proteomics with quantitative LC−MS/MS, MSI and pimonidazole-stained immunohistochemistry (IHC) sections to identify hypoxia-associated peptides in 4T1 tumor models^28^. Combining MSI with LC-MS/MS resulted in 18 identified proteins. Out of six selected proteins, the corresponding m/z values of five were statistically associated with the hypoxia pixels from IHC stainings. In their extensive analysis, also protein candidates from LC-MS/MS of microdissected hypoxia regions were identified. From these proteins, many are in line with our found candidates (e.g., LDHA, PGK1, LMNA, PKM, ANXA1, ALDOA). Mascini et al. pursued an indirect approach to detect hypoxia by identifying signals of the marker pimonidazole^26^. Besides being impractical as biomarker in a clinical setup, it might be that signals of pimonidazole obscure the actual molecular information of hypoxia itself.

From a methodological viewpoint, we also considered other machine learning methods and metrics for our research question. For example, a convolutional neural network (CNN) might as well have solved the task of predicting hypoxia from MSI data. However, if the primary task is to extract features, autoencoders are the better choice. In a CNN, it is not obvious which layer represents a good latent feature. Basic autoencoders may work well for conventional data, but miss spatial information in imaging data like MSI. Accordingly, convolutional autoencoders were found to exhibit better image compression and denoising abilities compared to vanilla autoencoders^29^. Denoising may be especially beneficial for spatial omics in general, which encounters variations in signal intensities across pixels. We also tested VAEs, commonly used for DR despite being primarily designed for generative purposes. Our results suggested that the mean latent representations of VAEs become overly general, lacking the specificity of hypoxia signals. On a similar note, we expect autoencoders which incorporate other regularization techniques or sparsity constraints to obscure low-abundant signals. Nevertheless, it may be worthwhile testing how these architectures perform in a semi-supervised setup, similarly to our semi-supervised ConvAE approach. Instead of autoencoders, other feature extraction methods may be used. Yet, autoencoders typically outperform linear DR in MSI analysis: Thomas et al. found that the features from PCA predominantly represent noise or trivial information^17^. In line with these findings, Matsuda et al. reported that their sparse autoencoder achieved better feature extraction results than PCA and comparable, sometimes better results than multivariate curve resolution^20^. Inglese et al. concluded that features from VAEs provide a more accurate representation of the tissue morphology than PCA^18^. Similarly, Gardner et al. argued that the features of PCA, maximum autocorrelation factors, and non-negative matrix factorization did not display strong spatial patterns when contrasted to their convolutional autoencoder.

Independent of the employed feature extraction method, the actual need for explainability and, consequently, a feature recovery method would have remained the same. Instead of our proposed recovery method to identify hypoxia features, we also considered to use SHAP values^30^. However, implementations of these algorithms usually assume feature independence^31^ and hence face analogous issues with highly correlated feature spaces as tree-based FI metrics. Regarding tree-based FI metrics, we considered both, impurity importance (IM) and permutation importance (PM). Though IM was considered to be biased towards categorical predictors^32^, this is not relevant for our purely continuous data. For correlated features, both metrics have their drawbacks. While IM were found to inflate independent features compared to features that correlate with one another^33^, PM overestimated the importance of correlated predictors^32^. Though all of these observations were derived from classification tasks, the same findings likely hold true for regression, given that the underlying algorithms remain consistent and only the impurity or model performance score is changing. Ultimately, we used IM, as our data lacks isolated independent variables, but rather features exhibiting different levels of correlation with other features. Our findings confirmed that IM mandates careful examination amidst high feature correlation. These observations were corroborated when feature redundancy was reduced considerably. However, we also showcased that convolutional autoencoders may companion RFs for robust biomarker discovery.

Several limitations need to be considered when interpreting the presented results. As shown in Table 1, one MSI mass might be assigned to several peptide candidates from tandem MS. Wrongly mapped MSI masses can be ascribed to the lower precision of m/z measurements acquired through TOF-based MS in comparison to other mass analyzers^2,3^. The lack of precision will gradually decrease in next-generation technologies that offer higher mass accuracy^34^, as was already demonstrated for FT-ICR MSI^35^. Independently from the mass analyzer used, our workflow ensures that the individual mass pairs are plausible by checking for high correlation in their ion images (Spearman correlation >0.8). Depending on the research question, the threshold can be increased for more stringent results. Alternatively, noisy associations may be eliminated with the semi-supervised ConvAE, at the expense of dismissing some true candidates. The choice between an unsupervised and a semi-supervised approach may therefore depend on the number of false positives and false negatives that can be accepted. Considering these limitations, our algorithm can provide peptide candidates from a set of MSI, tandem MS data along with some annotations. We plan to validate our peptide candidates further by using immunohistochemistry on consecutive tissue slices of the same samples.

Taken together, we showed how to retain low-abundant signals associated with hypoxia in MSI using convolutional autoencoders.

## Methods

### Animals and tumor models

The HNSCC xenograft models used in this work were part of a larger project on the effect of hypoxia with details of the animals and tumor models described in our pre-clinical study^36^. The animal facility and the experiments followed the ARRIVE guidelines and were approved according to the institutional guidelines and the German animal welfare regulations. The animals were sacrificed when the recurrent tumor reached the diameter of 15 mm or when the animal appeared to suffer. For tumor transplantation and biopsies, animals were anesthetized via i.p. injection of xylazine / ketamine (10 mg/kg and 100 mg/kg body weight, respectively). Animals were euthanized via cervical dislocation. A total of five samples of untreated tumors from the xenograft model CAL33 were utilized.

### Sample preparation and matrix-assisted laser desorption/ionization ions (MALDI) MSI protocol

Sections between 1 and 2 µm of tumors were cut from Formalin Fixed Paraffin Embedded (FFPE) tissue blocks. Sections were dewaxed and hydrated using a descending alcohol series (2x xylol; 100/96/70/50% ethanol, water each 5 min) and washed in water twice, heated in water at 110° for 20 min. and dried out in vacuum for at least 30 min. Proteins were digested with trypsin solved in 20 mM Ambic and covered with matrix (α-cyano4-hydroxycinnamic acid [CHCA]) solved in acetonitrile and TFA at 75 °C. Measurements were performed on Rapiflex Tissuetyper (Bruker) in positive reflector mode, calibrated by an external peptide mixture added next to the tissue sections (9 peptides, protein standard II, Bruker) and target flatness control on six marginal spots. Images were acquired with a raster width of 50 µm and with a mass range set between m/z 600 and 3200. A total of 53,400 m/z values were acquired for each spot, with a distance of 0.0487 between individual m/z values. A consecutive tissue slice (3 µm) was stained with anti-pimonidazole polyclonal antibody (PAb2627, Hypoxyprobe, Inc (HPI), Burlington, USA) diluted 1:100, followed by incubation with AF488-conjugated anti rabbit secondary antibody (A11034, Invitrogen, Thermo Fisher Scientific Inc., Massachusetts, USA), diluted 1:500, to visualize hypoxic regions.

### MSI pre-processing

All five MSI samples were measured individually, thus resulting in 53,400 m/z values which differ per sample. In order to derive a common set of peaks, several steps were applied (Supplementary Fig. 14): First, we calculated 53,400 mean m/z values based on all samples. Then we applied peak picking on the mean spectra of all samples individually, with a required mean signal-to-noise-ratio (SNR) of 6. Peaks not satisfying this condition were considered as noise and removed. The found peaks were then mapped back to the 53,400 mean m/z values using binary search, resulting in peaks of different samples but with a similar mass being mapped to the same mean m/z value bin. For peaks belonging to the same mean m/z value bin, their mean was calculated to derive the final peak references. This led to a total of 18,735 peaks. The spectra was normalized using their total-ion-count (TIC). Alternatively, the number of reference peaks can be further reduced by only considering bins with a minimum number of assignments, thereby summarizing mass shifts. This strategy is referred to as binning in MS, and is a common pre-processing practice to reduce mass shifts^37^. For paragraph *Relevance of high feature redundancy* in the results, this minimum number was set to 3 and 4, resulting in 2642 and 775 peaks respectively. If the minimum number of assignments to a bin is set to 1, mass shifts between different samples are explicitly not further corrected. Instead, multiple peaks belonging to the potential same mass are retained (Supplementary Fig. 15), expecting that the convolutional autoencoder will aggregate the peaks of these potential mass shifts into the same latent space feature(s). Staying close to the original measurements without correcting for mass shifts increases the probability to map masses of MSI measurements to the masses measured through tandem MS. This acknowledges the fact that the exact mass is unknown. This strategy lead to a total of 56 m/z values which can be considered true replicates, meaning that their intensity values are identical to another m/z value in all pixels. We estimated that around 320 more m/z values can be considered replicates, as their intensities among pixels are not identical but very similar (pearson correlation coefficient >0.975) to up to two neighboring m/z values. Intensities between samples were normalized with a global scaling factor per m/z value as described by ref. ^38^.

### Data characteristics

The MSI data processed exhibit the following characteristics. (1) Up to 4 raw m/z values represent isotopes and thus should be easily summarizable (see Supplementary Fig. 16). (2) During MSI pre-processing potential mass shifts are retained, leading to replicates (in total around 376 m/z values) that are likely to collapse in the same latent feature(s) of the autoencoder. (3) Due to the nature of trypsin, several m/z values are expected to belong to the same digested peptide. These m/z values are expected to show high correlations among each other. The high redundancy of data was considered when experimenting with different latent space configurations.

### Training of autoencoder

MSI data and hypoxia annotations, derived from consecutive slices that were stained for pimonidazole, were co-registered. The actual hypoxic spots were labeled by the same biologist in our team (M.J.B.) to avoid artificial hypoxic spots. The MSI data of 5 samples (including their hypoxia annotations) were split into 3 training and 2 validation samples. Intensities of MSI were normalized to a range between [0, 1]. Likewise, annotations were normalized to a range between [0, 1], whereas 0 indicates no hypoxia and 1 indicates the maximum hypoxia intensity found. All samples were then cut into patches of size 3 × 3 pixels. To increase the sample size, the autoencoder was trained on overlapping patches. Patches containing only background were discarded. With a step size of 2, this resulted in a total of 8649 patches for training and 2158 patches for validation. In the unsupervised mode the autoencoder was trained for 25 epochs and in the semi-supervised mode for 50 epochs in which also the hypoxia annotations were utilized. In both modes the Adam stochastic gradient optimizer with a learning rate of 1e-4 was used, with a higher learning rate (1e-3) leading to deterioration of results.

### Convolutional autoencoder

The proposed convolutional autoencoder takes an input of (x,x,y) (Fig. 2), where x denotes the patch size and y the number of features. All experiments were performed with a configuration of (x = 3, x = 3, y = 18,735). The first hidden layers consist of a convolutional layer with the following configuration: kernel size of 1; stride of 1; padding set ”valid”, followed by a BatchNormalization layer and a ReLU activation function. This reduces the dimensions to (3, 3, 1024). The second hidden layer consists of a convolutional layer which only differs from the first one by setting the kernel size to 2, followed again by a BatchNormalization layer, and a ReLu activation function. Here, the dimensions are reduced to (2,2*,z*^2^), denoted in the figure by b = 1. The final latent space size z^2^ was set to 8–256 in order to compare different configurations. Figure 2 sketches the encoder of the proposed autoencoder. The decoder was built symmetrically to the layers of the encoder. For the experiments shown in Supplementary Fig. 5, x was changed from 3 to 2, 4, and 6 accordingly, with all other parameters remaining fixed. For the experiments shown in Supplementary Figs. 6 and 7, the configuration was changed to x = 5, y = 18,735, z^1^ = 1024, b = 3 (i.e., kernel size = 4), z^2^ = 64 and x = 7, y = 18,735, z^1^ = 1024, b = 5 (i.e., kernel size = 6), z^2^ = 64, respectively. The autoencoder aims to learn an optimal encoding by minimizing the loss between the original and the reconstructed data. When optimizing for a minimal mean reconstruction error, this would favor reducing the error for intensities observed across many m/z values within various patches. We therefore applied an adjusted mean absolute error (MAE), which sums up the error for all intensities in all m/z values instead of taking the mean:

1 $${\rm{abs\; diff}}(k,j,j,i)={\rm{|}}{x}_{k,j,j,i}-{\hat{x}}_{k,j,j,i}{\rm{|}}$$

2 $${\rm{MAE}}\, {\rm{adj}}=\frac{1}{{batches}}\mathop{\sum }\limits_{k=1}^{{batches}}\frac{1}{{patches}}\mathop{\sum }\limits_{j=1}^{{patches}}\mathop{\sum }\limits_{i=1}^{{features}}{\rm{abs}}\, {\rm{diff}}_{k,j,j,i}$$

where *x* and $\hat{x}$ denote the actual and predicted intensity values respectively.

For the semi-supervised approach, an additional supervised error was calculated as follows:

3 $${\rm{supervised}}\, {\rm{error}}=\frac{1}{{pixels}}\mathop{\sum }\limits_{p=1}^{{pixels}}\mathop{\sum }\limits_{i=1}^{{features}}{\rm{abs}}\, {\rm{diff}}\, {\rm{selected}}_{p,i}$$

whereas the supervised_error is only calculated for pixels with a certain degree of hypoxia (>0.6). The autoencoder in the semi-supervised mode will then minimize the sum of *MAE adj* and *supervised error*.

For the convolutional variational autoencoder (ConvVAE) approach, the negative Kullback-Leibler (KL) divergence between the learned distribution and the standard Gaussian distribution was minimized as follows^39^:

4 $${\text{KL}}=-0.5\cdot \mathop{\sum }\limits_{i=1}^{n}\left(1+\log \left({\sigma }_{i}^{2}\right)-{\mu }_{i}^{2}-{\sigma }_{i}^{2}\right)$$

where *n* represents the number of latent space dimensions, and µ_i_ and σ_i_^2^ represent the mean and the variance of the *i*-th dimension of the latent space, respectively. Additionally, the adjusted MAE was used as reconstruction error. In the variational approach, the autoencoder will minimize the sum of *MAE adj* and *KL*. The overall architecture and configuration of the non-variational convolutional autoencoder was employed (x = 3, y = 18,735, z^1^ = 1024, b = 1, z^2^ = 64), followed by the variational-specific layers (i.e., computation of mean and log variance, sampling). The final latent space size was set to z^2^ as defined above.

### Training of RF regression on hypoxia annotations

Random forest regression models were trained to predict the degree of hypoxia (range between [0, 1]) in a patch with the following parameters: 1000 trees, *max_features/mtry* being set to the square root of the total number of features. For 2 of the 5 samples, the number of hypoxic spots were too low, such that they were discarded for the regression task. For every annotation patch, the mean value was calculated to derive the required input shape for the random forest regression models. In the convolutional autoencoder (ConvAE) approach, the mean of every encoded MSI patch (2 × 2 pixels) was calculated whereas in the RF only approach, the mean of the original MSI patch was calculated instead. The random forest regression model was trained with overlapping patches using a step size of 1. Additionally, non-hypoxia patches were downsampled to balance the number of hypoxic and non-hypoxic patches, resulting in a total of 6409 patches. The individual m/z values (RF only approach) or latent m/z values (ConvAE approach) were then ranked according to their feature importance for hypoxia (based on 10-fold cross validation) based on their mean decrease in impurity (using mean squared error).

### Tandem MS

To derive actual peptides from m/z values of MSI experiments, additional tandem mass spectrometry were carried out. Three samples from the same tumor model were used to derive peptide information from tandem MS using FFPE samples. Protein extraction from FFPE tissues was carried out by combining 3 sections with 15 µm thickness for each sample. The FFPE Qproteome Kit (Qiagen, Catalog No. 37623, Germany) was used for protein extraction and purification following the manufacturer’s instructions with minor modifications. All protein lysates were precipitated with 4 volumes of ice-cold acetone and protein pellets were resuspended in 20 µl of Laemmli sample buffer. In-Gel digestion of the samples and MS experiment and analysis were performed at the Proteomics core facility (DKFZ, Heidelberg).

Proteins from FFPE slices were run for 0.5 cm into an SDS-PAGE and the entire piece was cut out and digested using trypsin according to ref. ^40^. adapted to on a DigestPro MSi robotic system (INTAVIS Bioanalytical Instruments AG).

The LC-MS/MS analysis was carried out on an Ultimate 3000 UPLC system (Thermo Fisher Scientific) directly connected to an Orbitrap Exploris 480 mass spectrometer for a total of 150 min. Peptides were online desalted on a trapping cartridge (Acclaim PepMap300 C18, 5 µm, 300 Å wide pore; Thermo Fisher Scientific) for 3 min using 30 ul/min flow of 0.05% TFA (v/v) in water. The analytical multistep gradient (300 nl/min) was performed using a nanoEase MZ Peptide analytical column (300 Å, 1.7 µm, 75 µm × 200 mm, Waters) using solvent A (0.1% formic acid (v/v) in water) and solvent B (0.1% formic acid (v/v) in acetonitrile). For 132 min the concentration of B was linearly ramped from 4% to 30%, followed by a quick ramp to 78%, after two minutes the concentration of B was lowered to 2% and a 10 min equilibration step appended. Eluting peptides were analyzed in the mass spectrometer using data depend acquisition (DDA) mode. A full scan at 120 k resolution (380–1400 m/z, 300% AGC target, 45 ms maxIT) was followed by up to 2 s of MS/MS scans. Peptide features were isolated with a window of 1.4 m/z, fragmented using 26% NCE. Fragment spectra were recorded at 15 k resolution (100% AGC target, 54 ms maxIT). Unassigned and singly charged eluting features were excluded from fragmentation and dynamic exclusion was set to 35 s.

Data analysis was carried out by MaxQuant (version 1.6.14.0^41^) using an organism specific database extracted from Uniprot.org under default settings (human containing 79,038 entries from 03.01.2022). Identification false discovery rate (FDR) cutoffs were 0.01 on peptide level and 0.01 on protein level. Match between runs option was disabled. For quantification iBAQ-values^42^ and a label free quantification approach based on the MaxLFQ algorithm^43^ was applied. A minimum of 2 quantified peptides per protein was required for LFQ protein quantification.

In total 28487 peptides and 3160 proteins could have been identified by tandem MS based on an FDR cutoff of 0.01 on peptide level and 0.01 on protein level. Identified in all samples were 8509 peptides and 2254 proteins. A total of 3114 proteins could have been quantified, of which 1255 were quantified in all samples.

## Mapping of MSI masses to tandem MS masses

The m/z values from MSI were transformed to masses using the formula

5 $${\rm{MSI\; mass}}={\rm{m}}/{\rm{z\; value}}* 1-1$$

given that the majority of matrix-assisted laser desorption/ionization ions tend to be single charged^3^.

Modified peptides from tandem M/S were excluded from further analysis, indicated by a ”C” in the sequence. Given that for MSI, the true mass is unknown, we assumed that the full width at half maximum (FWHM) approximates the actual mass range. This assumption needs to be adjusted depending on the instrument’s specificity (e.g., calibration, resolution, among others). Accordingly, the FWHM was calculated for every peak and sample individually. We then defined the standard error as the range of the FWHM of all samples. The *lower half-maximum point*, respectively *upper half-maximum point* is the point left respectively right to a peak where it reaches half of its maximum. Additionally, a minimal technical error was defined as the distance between two m/z values, i.e., 0.0487. The *lower technical point*, respectively *upper technical point* is then defined as the peak - 0.0487, respectively peak + 0.0487.

A given MS/MS mass was matched with a given MSI mass if the following condition was fulfilled:

6 $${\rm{MSI}}\; {\rm{mass}}\min \,< \,={\rm{MS}}/{\rm{MS}}\; {\rm{mass}} \,< \,={\rm{MSI}}\; {\rm{mass}}\max$$

whereas

7 $$\begin{array}{c}{\rm{MSI}}\; {\rm{mass}}\,{\mathrm{min}}=\max (\mathop{\rm{min}}\limits_{i=1}^{n}\,{{\rm{lower}}\; {\rm{half}}\;{\rm{maximum}}\; {\rm{points}}}_{{{\rm{sample}}}_{i}},{\rm{lower}}\; {\rm{technical}}\; {\rm{point}})\\ {\rm{MSI}}\; {\rm{mass}}\,{\mathrm{max}}=\min (\mathop{\max}\limits_{i=1}^{n}{{\rm{upper}}\; {\rm{half}}\;{\rm{maximum}}\; {\rm{points}}}_{{{\rm{sample}}}_{i}},{\rm{upper}}\; {\rm{technical}}\; {\rm{point}})\end{array}$$

and *n* denotes the number of samples.

The effect of these two errors is depicted in Supplementary Fig. 17. Supplementary Fig. 17a, b show two neighboring peaks, likely denoting mass shifts, that share the same standard error boundaries. However, while the minimal error is further limited by the standard error in Supplementary Fig. 17a, this is not the case in Supplementary Fig. 17b. Using this combined error to limit the ranges of *MSI mass min* and *MSI mass max* is therefore especially conservative if peaks are not backed up by mass shifts (e.g., Supplementary Fig. 17c). Only those MSI masses where matched, which were associated with hypoxia. Peptides were only considered as candidates if at least two distinct MSI masses (without counting potential mass shifts) could be matched. In addition, the ion images of two mass pairs are expected to correlate with one another (Spearman correlation coefficient >0.80).

### Software

For MSI pre-processing, R (4.1.0) and the Cardinal (2.10.0) package were utilized^44^. Image co-registration was performed with a similarity transform (i.e., affine transformation without sheering) using the ITKElastix (0.17.1)^45,46^ framework for Python. Therefore, images stained for pimonidazole were downsampled to the spatial resolution of MSI. The convolutional autoencoder and downstream analysis were developed using Python (3.8.8) and Tensorflow (2.12.0). The RF models were built using sklearn (1.3.0). Boxplots were created with statannotations (0.6.0)^47^ and seaborn (0.11.2)^48^, with statistical tests and multiple test correction being performed in SciPy (1.11.4)^49^ in Python (3.9.18).

### Statistical tests and visualization

For all statistical tests, two-sided Mann–Whitney U rank tests were conducted. SSIM scores were compared either among different approaches (e.g., RF only versus unsupervised ConvAE), configurations (e.g., patch size 3 versus patch size 5) or between latent features of the ConvAE approaches (e.g., latent feature 56 versus latent feature 37). The hypothesis being tested was that the distribution of SSIM scores for one approach/configuration/latent feature differs stochastically from another approach/configuration/latent feature. To illustrate that the poorer performance of an approach (e.g., RF) is not attributable purely due to a higher number of features, Mann–Whitney U rank tests were also performed to compare the number of features being retrieved among multiple runs (e.g., RF only approach against unsupervised ConvAE approach). Similarly, the unsupervised ConvAE latent space configurations were tested, with the hypothesis that the distribution of the R^2^ adjusted scores differs significantly between a latent space size of 64 and all other configurations. False discovery rate at 5% were controlled using the Benjamini and Hochberg method^50^. Adjusted values of *p* < 0.05 were considered statistically significant. Metrics were visualized using boxplots following the standard Tukey representations. Boxes represent the interquartile range (IQR), with the horizontal line indicating the median value. Whiskers indicate the largest (respectively smallest) value within 1.5 times the IQR above the 75th (respectively below the 25th) percentile.

### Reporting summary

Further information on research design is available in the Nature Research Reporting Summary linked to this article.

### Supplementary information

Supplementary information

Reporting Summary

Supplementary table - peptide candidates from unsupervised approach

Supplementary table - peptide candidates from semi-supervised approach

## Data Availability

The mass spectrometry proteomics data (both, MSI and LC-MS/MS) have been deposited to the ProteomeXchange Consortium via the PRIDE^51^ partner repository with the dataset identifier PXD047820.

## Code availability

The source code is publicly available on GitHub: https://github.com/DKFZ-ABI/automsi.
