# Supplementary material for: Enhancing mass spectrometry imaging accessibility using convolutional autoencoders for deriving hypoxia-associated peptides from tumors
Source: NPJ Syst Biol Appl. 2024 May 27;10:57. doi: 10.1038/s41540-024-00385-x (PMC11130291; doi:10.1038/s41540-024-00385-x)
Supplement: Supplementary file 1 — Supplementary information [file 41540_2024_385_MOESM1_ESM.pdf]

Supplementary information

Enhancing mass spectrometry imaging accessibility using convolutional autoencoders for deriving hypoxia-associated peptides from tumors

Corresponding Author: Verena Bitto, German Cancer Research Center (DKFZ), Heidelberg, Germany.

Supplementary discussion

Importance of patch size and kernel size

Considering the same autoencoder configuration as used throughout the paper (x = 3, y = 18,735, $z^1 = 1024$, b = 1, $z^2 = 64$), x is adjusted in the following to show the effect of an adjusted patch size.

As shown in Supplementary Figure 5, a change in patch size is altering the latent representations only moderately. Of note, the precise latent representations, like the overall contrast, differ in every autoencoder run independently of the patch size. A patch size of 2×2 pixels (Supplementary Figure 5a) impedes the kernels in the second layer to traverse the input patches, reflected by visible squares. A patch size of 1×1 would effectively disable the possibility to incorporate the spatial context of the data and would be incompatible with the used kernel size of 2×2 pixels of the proposed architecture. In contrast, a higher patch size will allow the kernels to traverse larger areas, for the sake of being less memory efficient. Supplementary Figures 5b and 5c show that an increased patch size leads also to an increased size of the latent representations. Supplementary Table 2 shows the size dependency of the latent representation to the input patch size, whereas

$$\text{Latent space dimension (px)} = \text{No. of patches per dimension} \times (\text{Patch size} - 1)$$

The patch size is subtracted by one, as the kernel size of 2×2 pixels reduces the spatial dimensions of the patches accordingly.

Closely related to the patch size, the kernel size influences which area the autoencoder focuses on during feature extraction. Considering that the hypoxia regions in the samples under investigation do not spread over large regions, they are likely to be missed with an increased kernel size. Supplementary Figures 6a–6b illustrate two exemplary latent features each in two unsupervised convolutional autoencoder (ConvAE) runs when the patch size and kernel size were increased to 5 and 4 (latent space configuration: x = 5, y = 18,735, z^1 = 1024, b = 3 (i.e., kernel size = 4), z^2 = 64) or 7 and 6 (latent space configuration: x = 7, y = 18,735, z^1 = 1024, b = 5 (i.e., kernel size = 6), z^2 = 64), respectively. Only the kernel size of the second hidden layer of the autoencoder was adjusted. The latent features on the left hand side of the figure show the features with the highest feature importance for hypoxia and the one on the right hand side one additional latent feature to highlight that the autoencoder focuses more on larger structures. For the hypoxia-associated latent features, the recovery method revealed a total of 466 contributing mass-to-charge (m/z) values for the run with a kernel size of 4, and 11 contributing m/z values for the run with a kernel size of 6. The number of derived features already suggests that the latent features are either too unspecific, resulting in too many associations, or too abstract, resulting in only few associations. Supplementary Figure 6c shows the structural similarity index measure (SSIM) scores of both runs compared to the exemplary run of the unsupervised ConvAE approach with a patch size of 3 and a kernel size of 2. Of note, some individual runs with a kernel size of 4 were able to achieve similar or even slightly better SSIM scores than the exemplary run with a kernel size of 2 (Supplementary Figure 7). However overall, results obtained with a higher kernel size exhibited lower SSIM scores and were generally more diverse and less deterministic.

Besides the training of the autoencoder, the patch size has a greater influence on the performance on the random forest (RF) regression models. Supplementary Figure 8 shows that a higher patch size results in a greater number of pixels being considered when deriving the mean hypoxia value. As a consequence, the regression model can no longer account for the precise position of particular small hypoxic spots. This effect deteriorates the performance of the RF especially when training is carried out on fewer overlapping patches. The number of overlapping patches is defined by the step size, i.e., a step size of 1 generates the maximum amount of overlapping patches. To assess the impact, the topmost-ranked features were compared to the reference

mass-to-charge (m/z) value 998.472 at a patch size of 3 and 5, with a fixed step size of either 1 or 2 in 10 RF only runs, considering all cross-validation results. This comparison takes into account that a change in the step size will influence the total number of patches for training the RF. Supplementary Figure 9 shows that among the topmost-ranked features, more outliers with a low SSIM were picked up by the RF at a patch size of 5 compared to a patch size of 3, given the step size was set to 1. At a step size of 2, the SSIM score of experiments with a patch size of 5 deteriorated further in comparison to runs with a patch size of 3.

Convolutional variational autoencoder (ConvVAE)

In a ConvVAE, the encoder is outputting the mean and variance of a latent space dimension to describe its distribution. This is different to a non-variational approach, in which the encoder outputs a direct mapping from the input. For comparison, the overall architecture and configuration of the non-variational approach was employed (see Methods for details). After training the ConvVAE, mean latent representations were extracted to train the RF regression model and subsequently ranking them for association with hypoxia. Supplementary Figure 10 shows exemplary mean latent space representations of the ConvVAE from different runs, with the top-ranked hypoxia-associated feature shown on the left hand side and one additional feature on the right hand side. When comparing the recovered m/z values to the reference m/z value 998.472, the SSIM scores of the ConvVAE were found to be significantly lower than those of the proposed ConvAE (Supplementary Figure 11). This suggests that the mean latent features of the ConvVAE do not retain sufficient hypoxia information.

Redundancy of m/z values

Supplementary Figures 12 and 13 illustrate that even when the number of m/z values is drastically reduced (from 18,735 to 2,642 and 775 m/z values, respectively), the SSIM scores of the ConvAE approaches are higher than those of RFs alone. Due to the difference in pre-processing the samples (see Methods), the reference m/z value changed from 998.472 (18,735 m/z values) to 998.502 (2,642 m/z values) and 999.4807 (775 m/z values), respectively.

Supplementary figures

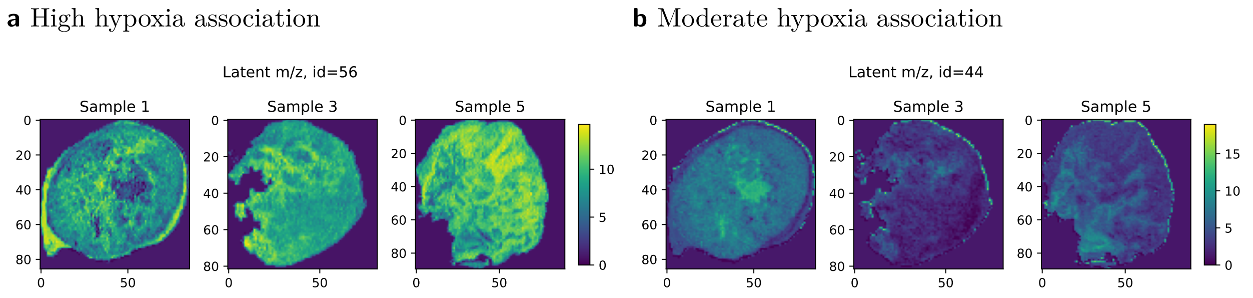

Supplementary Figure 1: Representation of individual samples in latent space. a Latent feature with highest associated to hypoxia annotations according to random forest feature importance. **b** Latent feature with moderate hypoxia association according to random forest feature importance.

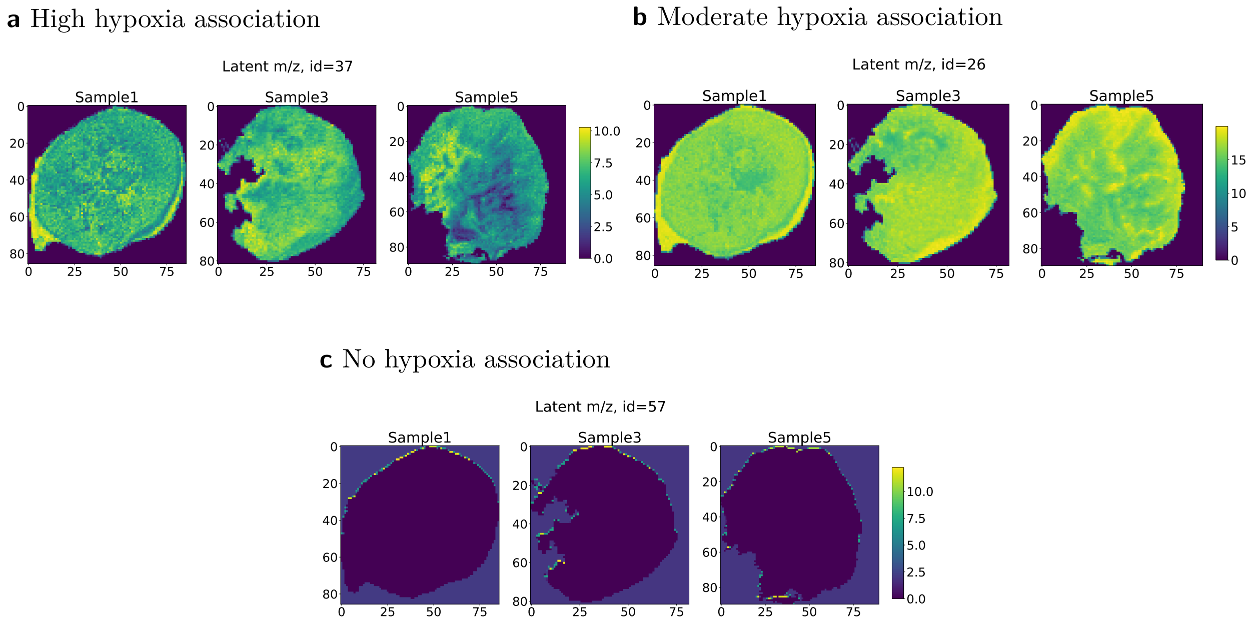

Supplementary Figure 2: Additional representations of latent features with diverse scores for hypoxia association. a High hypoxia association, **b** moderate hypoxia association and **c** no hypoxia association according to random forest feature importance.

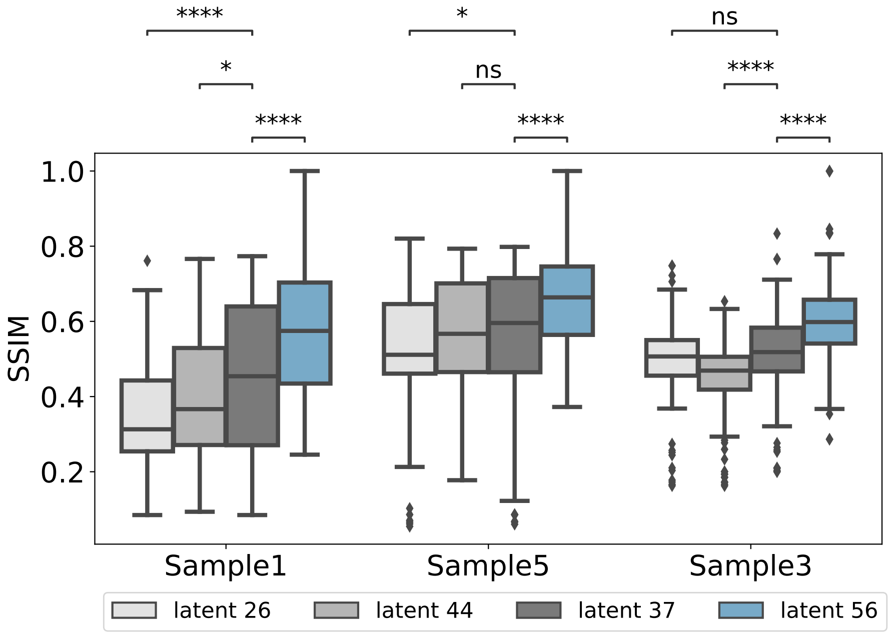

Supplementary Figure 3: Structural similarity index measure (SSIM) of mass-to-charge (m/z) values recovered from different latent features to the reference m/z value 998.472 in exemplary unsupervised convolutional autoencoder (ConvAE) run. Latent feature #26 and #44 were found to be moderately associated with the hypoxia annotations. Latent feature #37 achieved the second highest feature importance score and latent feature #56 was ranked highest. Higher scores denote higher similarity to the reference m/z value 998.472. Boxplots follow the Tukey style (see Methods), incorporating p value cutpoints: **** $< 10^{-4}$, *** < 0.001, ** < 0.01, * < 0.05, ns $>= 0.05$. Groups were compared using two-sided Mann-Whitney U rank tests, where p values were corrected to control the false discovery rate.

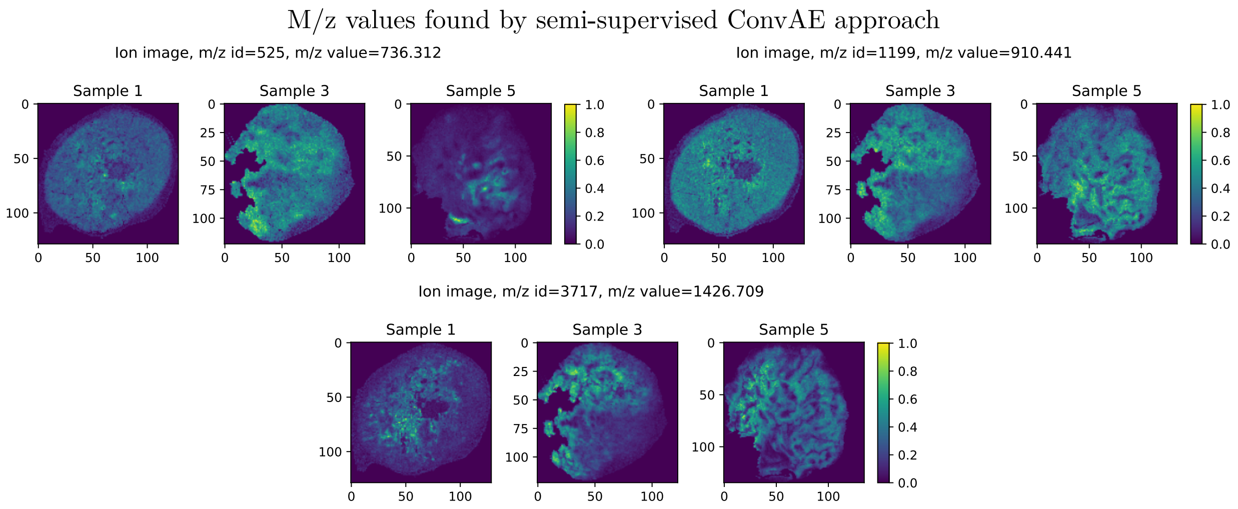

Supplementary Figure 4: Exemplary mass-to-charge (m/z) values that were distinctively associated with hypoxia by the semi-supervised convolutional autoencoder (ConvAE) approach.

a Patch size x = 2, kernel size = 2

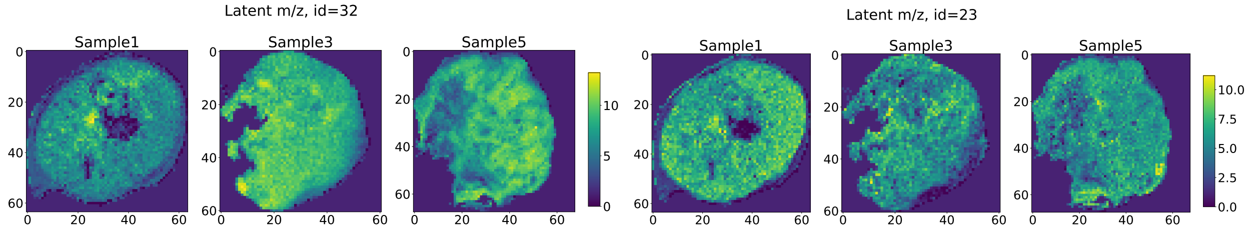

b Patch size x = 4, kernel size = 2

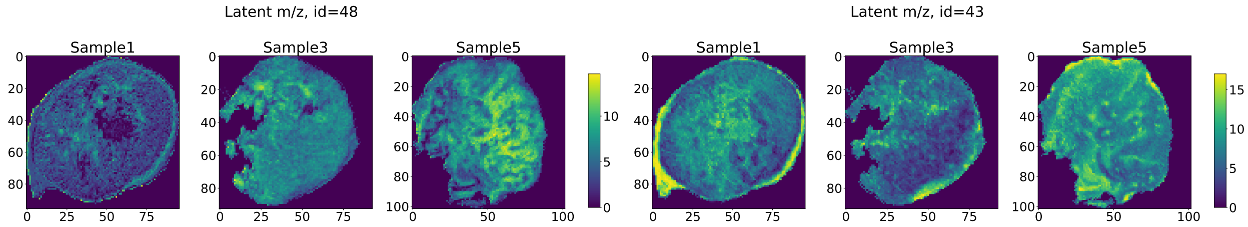

c Patch size x = 5, kernel size = 2

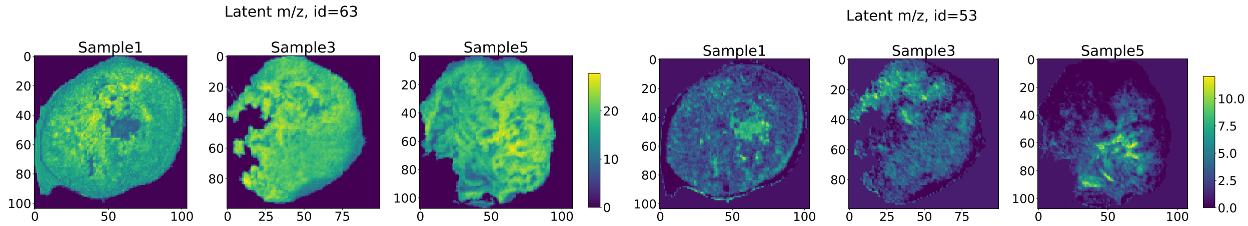

Supplementary Figure 5: Effect of adjusted input patch size in unsupervised convolutional autoencoder (ConvAE) runs. Apart from the patch size, all other parameters remained fixed. Shown are latent features which exhibited the highest feature importance score for hypoxia (left) and one other latent feature (right) in different autoencoder runs with **a** patch size x = 2, **b** patch size x = 4 **c**, patch size x = 5. Latent representations depicted similar characteristics independent of the patch size. A higher input patch size increases the latent image size.

a Patch size x = 5, kernel size = 4

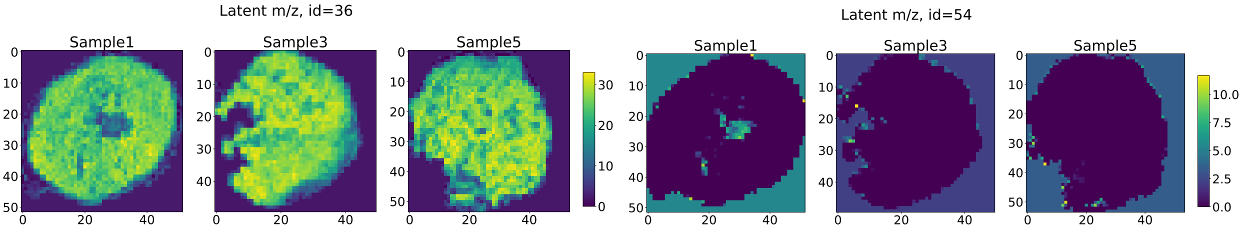

b Patch size x = 7, kernel size = 6

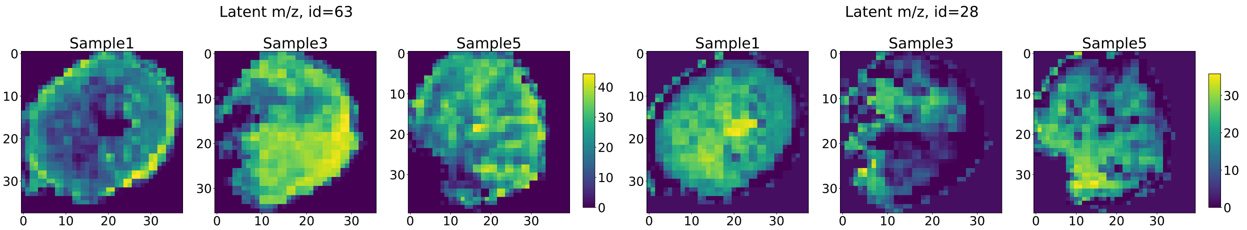

c Similarity scores of exemplary runs

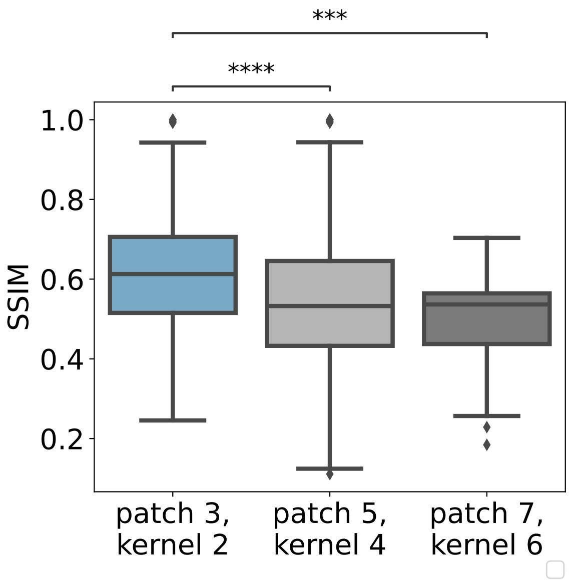

Supplementary Figure 6: Effect of adjusted kernel and patch size in unsupervised convolutional autoencoder (ConvAE) runs. Apart from the kernel size and the patch size, all other parameters remained fixed. **a, b** Shown are latent features which exhibited the highest feature importance score for hypoxia (left) and one other latent feature (right) in different autoencoder runs with **a** patch size x = 5, kernel size = 4 and **b** patch size x = 7, kernel size = 6. A higher input patch size increases the latent image size, while a higher kernel size decreases it. **c** Structural similarity index measure (SSIM) of all identified hypoxia-associated features to the reference mass-to-charge (m/z) value 998.472 in exemplary runs with adjusted kernel sizes and exemplary unsupervised ConvAE run shown in Fig. 6. Boxplots follow the Tukey style (see Methods), incorporating p value cutpoints: **** $< 10^{-4}$, *** < 0.001, ** < 0.01, * < 0.05, ns $>= 0.05$. Groups were compared using two-sided Mann-Whitney U rank tests, where p values were corrected to control the false discovery rate.

a Patch size x = 5, kernel size = 4

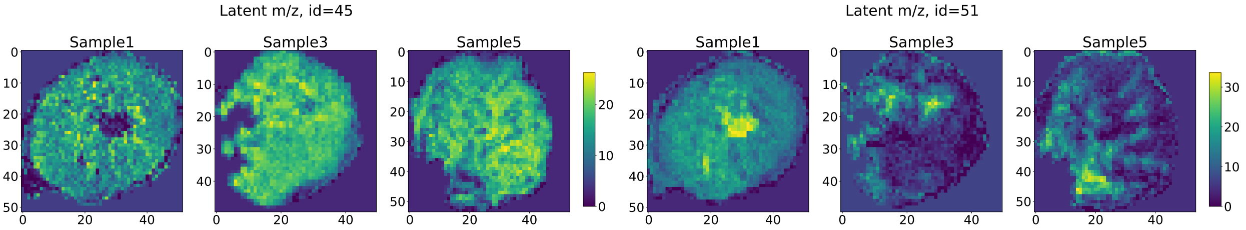

b Similarity scores of exemplary runs

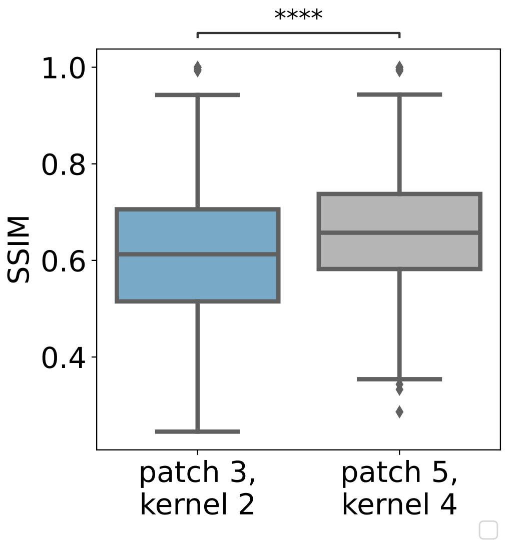

Supplementary Figure 7: Example in which an adjusted kernel and patch size in one unsupervised convolutional autoencoder (ConvAE) run retained hypoxia-associated features according to the structural similarity index measure (SSIM). Apart from the kernel size and the patch size, all other parameters remained fixed. **a** Shown are latent features which exhibited the highest feature importance score for hypoxia (left) and one other latent feature (right) in an additional autoencoder run with patch size x = 5, kernel size = 4. A higher input patch size increases the latent image size, while a higher kernel size decreases it. **b** SSIM of all identified hypoxia-associated features to the reference mass-to-charge (m/z) value 998.472 in exemplary run with adjusted kernel size and exemplary unsupervised ConvAE run shown in Fig. 6. Boxplots follow the Tukey style (see Methods), incorporating p value cutpoints: **** $< 10^{-4}$, *** < 0.001, ** < 0.01, * < 0.05, ns $>= 0.05$. Groups were compared using two-sided Mann-Whitney U rank tests, where p values were corrected to control the false discovery rate.

a Hypoxia annotations, patch size x = 3

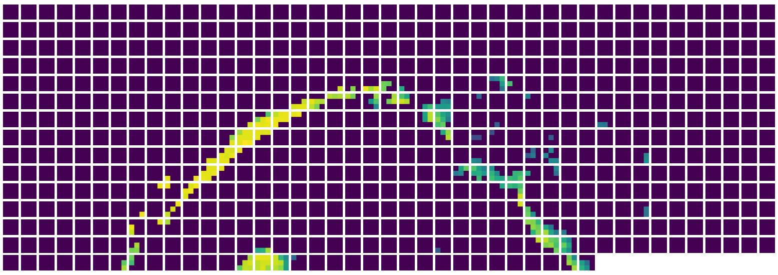

b Hypoxia annotations, patch size x = 5

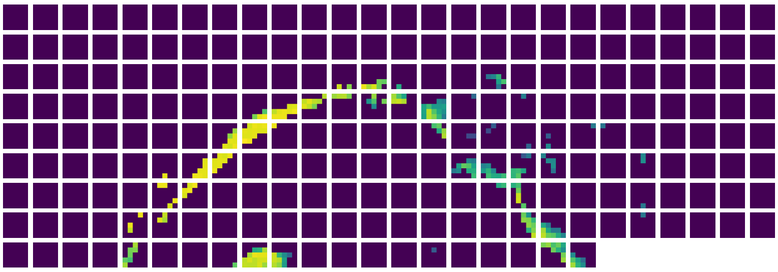

Supplementary Figure 8: Effect of increasing the patch size on annotation patches. Shown are some exemplary annotation patches of Sample 1. **a** Proposed patch size, x = 3, **b** increased patch size, x = 5 for the hypoxia annotations. The precise position of smaller hypoxic spots is obscured in larger patch sizes when using the mean values for summarization.

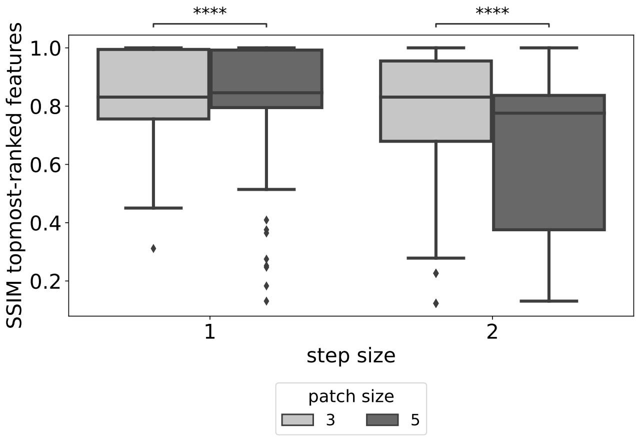

Supplementary Figure 9: Effect of increased patch size size on random forest (RF) regression models trained on overlapping patches. Shown is the structural similarity index measure (SSIM) between the topmost-ranked features to the reference mass-to-charge (m/z) value 998.472 with varying patch sizes at a step size of either 1 (left-hand side of image) creating the maximum number of overlapping patches, or 2 (right-hand side of image) creating a reduced set of overlapping patches, in all three samples and cross validation results of 10 RF only runs. Higher scores denote higher similarity to the reference m/z value 998.472. Boxplots follow the Tukey style (see Methods), incorporating p value cutpoints: **** $< 10^{-4}$, *** < 0.001, ** < 0.01, * < 0.05, ns $>= 0.05$. Groups were compared using two-sided Mann-Whitney U rank tests, where p values were corrected to control the false discovery rate.

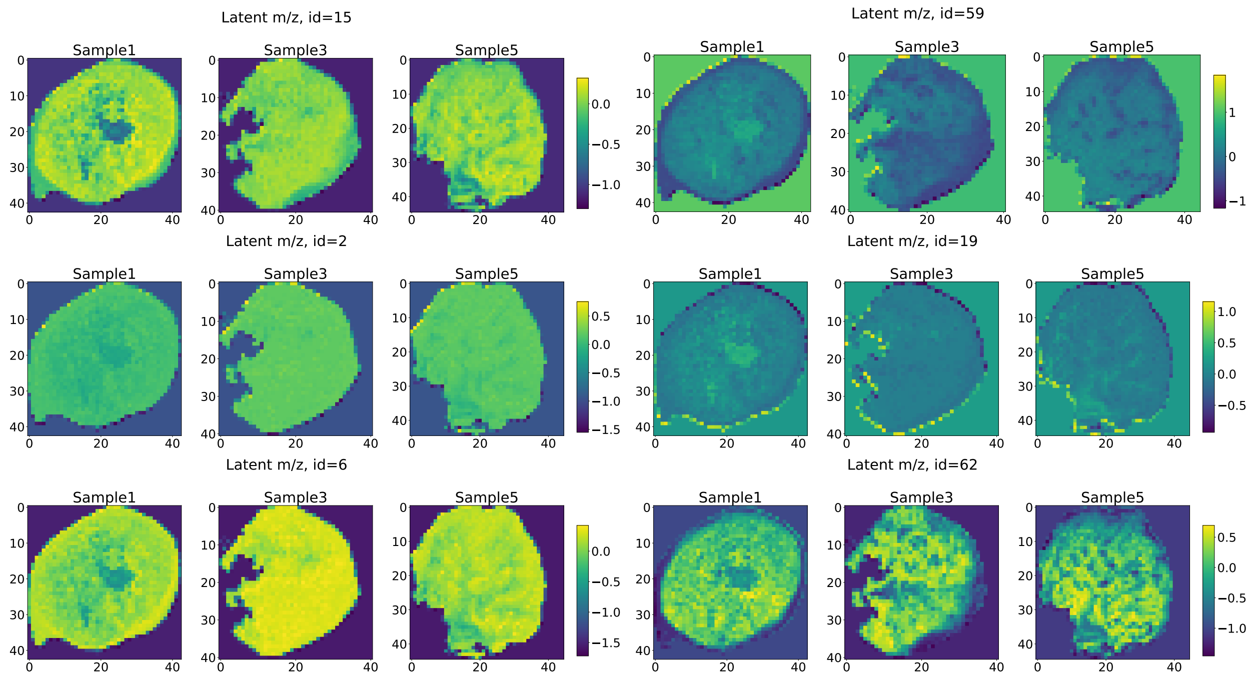

Supplementary Figure 10: Examples of mean latent features from different convolutional variational autoencoder (ConvVAE) runs. Shown are mean latent features which exhibited the highest feature importance score for hypoxia (left) and one other latent feature (right).

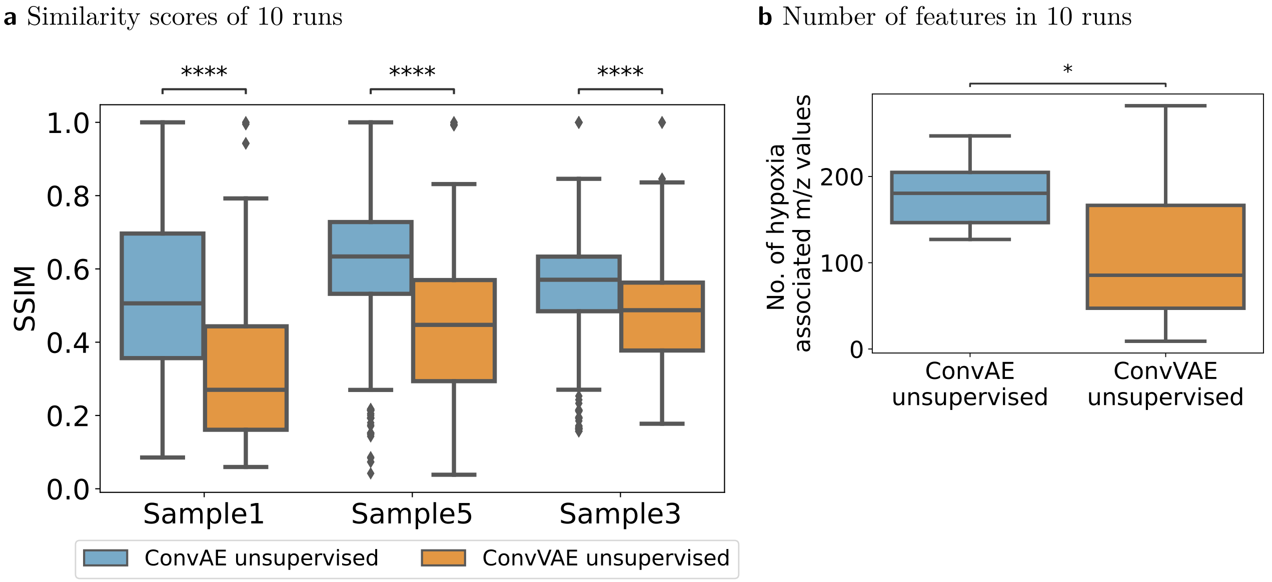

Supplementary Figure 11: Quantitative analysis of 10 runs each using the proposed non-variational unsupervised convolutional autoencoder (ConvAE) shown in Fig. 7b versus a unsupervised convolutional variational autoencoder (ConvVAE) approach. Boxplots follow the Tukey style (see Methods), incorporating p value cutpoints: **** $< 10^{-4}$, *** < 0.001, ** < 0.01, * < 0.05, ns $>= 0.05$. Groups were compared using two-sided Mann-Whitney U rank tests, where p values were corrected to control the false discovery rate. **a** Structural similarity index measure (SSIM) of all identified hypoxia-associated features to the reference mass-to-charge (m/z) value 998.472 per sample in 10 individual runs each. **c** Number of hypoxia-associated m/z values that were identified by both approaches in 10 runs.

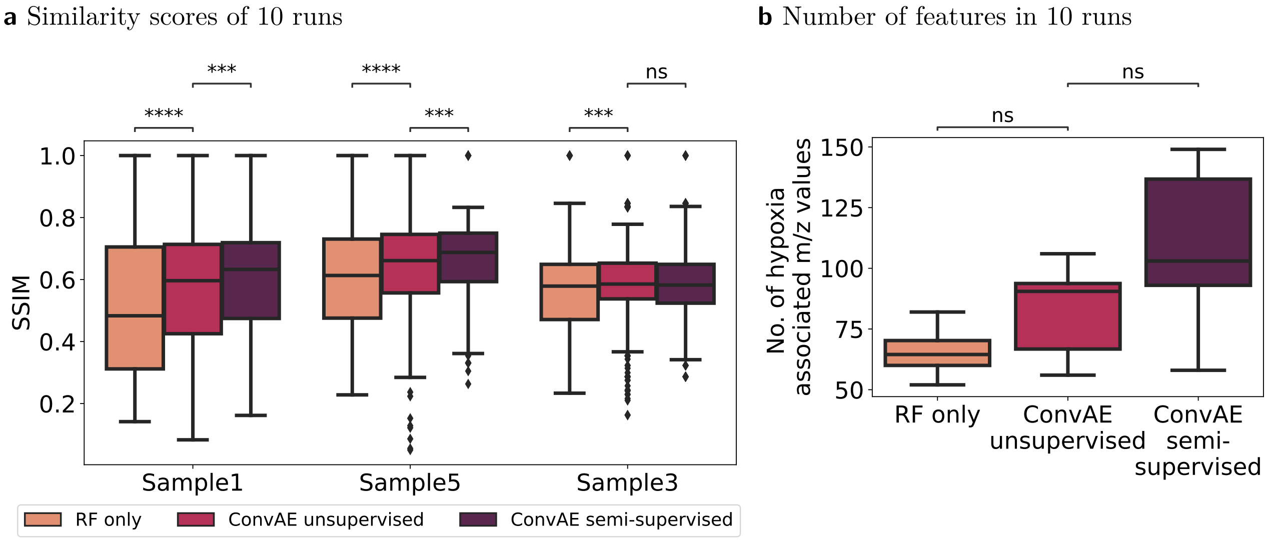

Supplementary Figure 12: Quantitative analysis of 10 runs each of convolutional autoencoder (ConvAE) and random forest (RF) only approaches, with the total number of features being reduced to 2,642 m/z values. Boxplots follow the Tukey style (see Methods), incorporating p value cutpoints: **** $< 10^{-4}$, *** < 0.001, ** < 0.01, * < 0.05, ns $>= 0.05$. Groups were compared using two-sided Mann-Whitney U rank tests, where p values were corrected to control the false discovery rate. **a** Structural similarity index measure (SSIM) of all identified hypoxia-associated features to the reference mass-to-charge (m/z) value 998.502 per sample in 10 individual runs each. **c** Number of hypoxia-associated m/z values that were identified by the three approaches in 10 runs.

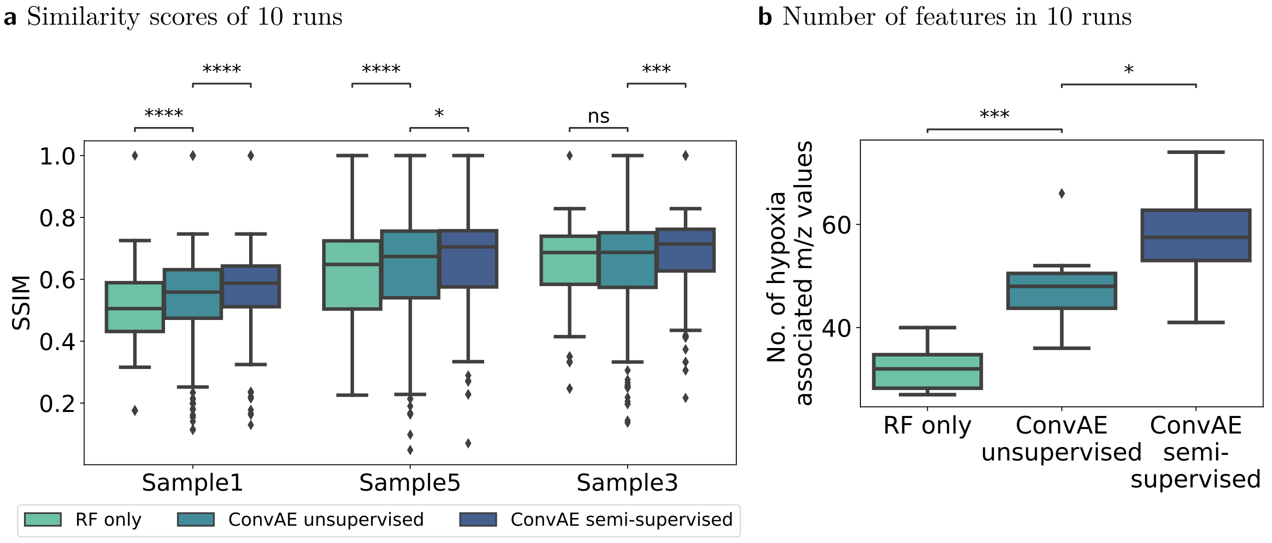

Supplementary Figure 13: Quantitative analysis of 10 runs each of convolutional autoencoder (ConvAE) and random forest (RF) only approaches, with the total number of features being reduced to 775 m/z values. Boxplots follow the Tukey style (see Methods), incorporating p value cutpoints: **** $< 10^{-4}$, *** < 0.001, ** < 0.01, * < 0.05, ns $>= 0.05$. Groups were compared using two-sided Mann-Whitney U rank tests, where p values were corrected to control the false discovery rate. **a** Structural similarity index measure (SSIM) of all identified hypoxia-associated features to the reference mass-to-charge (m/z) value 999.4807 per sample in 10 individual runs each. **c** Number of hypoxia-associated m/z values that were identified by the three approaches in 10 runs.

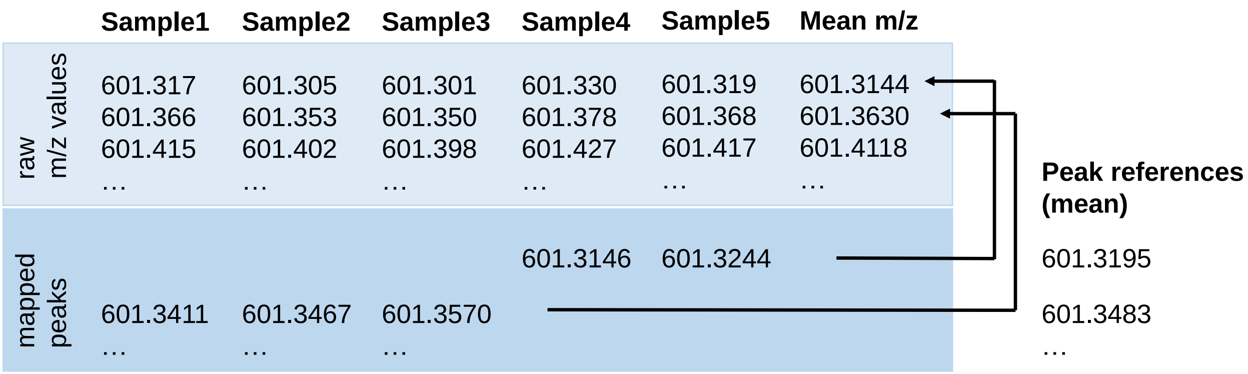

Supplementary Figure 14: Deriving peak references from multiple samples. First, mass-to-charge (m/z) values of all samples are sorted to define a set of mean m/z values. Second, peak picking on the mean spectra per sample is applied (not shown) and peaks mapped to the mean m/z values. M/z values from different samples but similar masses (e.g., 601.3146, 601.3244) will map to the same mean m/z value. These groups are utilized to derive peak references by taking their mean m/z value (e.g., 601.3195 from peaks 601.3146 and 601.3244).

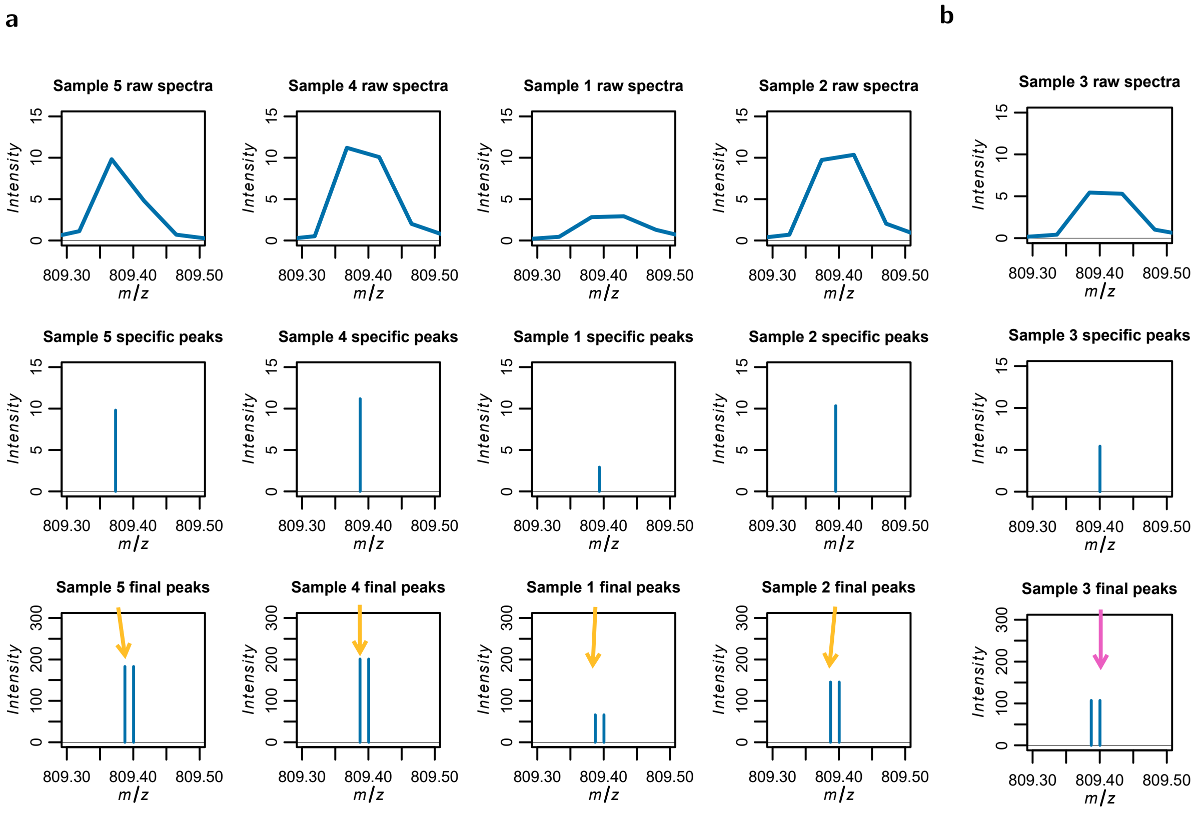

Supplementary Figure 15: Peak picking on multiple samples. Shown is the mean intensity of all pixels in the range of mass-to-charge (m/z) values 809.30 to 809.50 per sample. Row #1 shows the mean raw spectra per sample. Row #2 shows the peaks derived from the mean spectra of a given sample. Row #3 illustrates the final TIC-normalized peaks for all samples. **a** For Samples 5, 4, 1 and 2, their sample-specific peaks match best with mean m/z value 809.387. **b** For Sample 3, its sample specific peaks match best with mean m/z value 809.400. The two peaks around 809.387 and 809.400 are likely to denote mass shifts. As the true mass is unknown, both peaks are kept for further analysis.

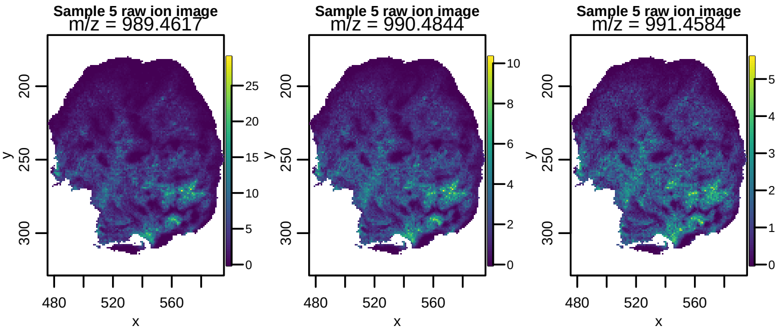

Supplementary Figure 16: Mass-to-charge (m/z) values (989.4617, 990.4844, 991.4584) representing isotopes from the same peptide. Shown are non-normalized ion images of Sample 5.

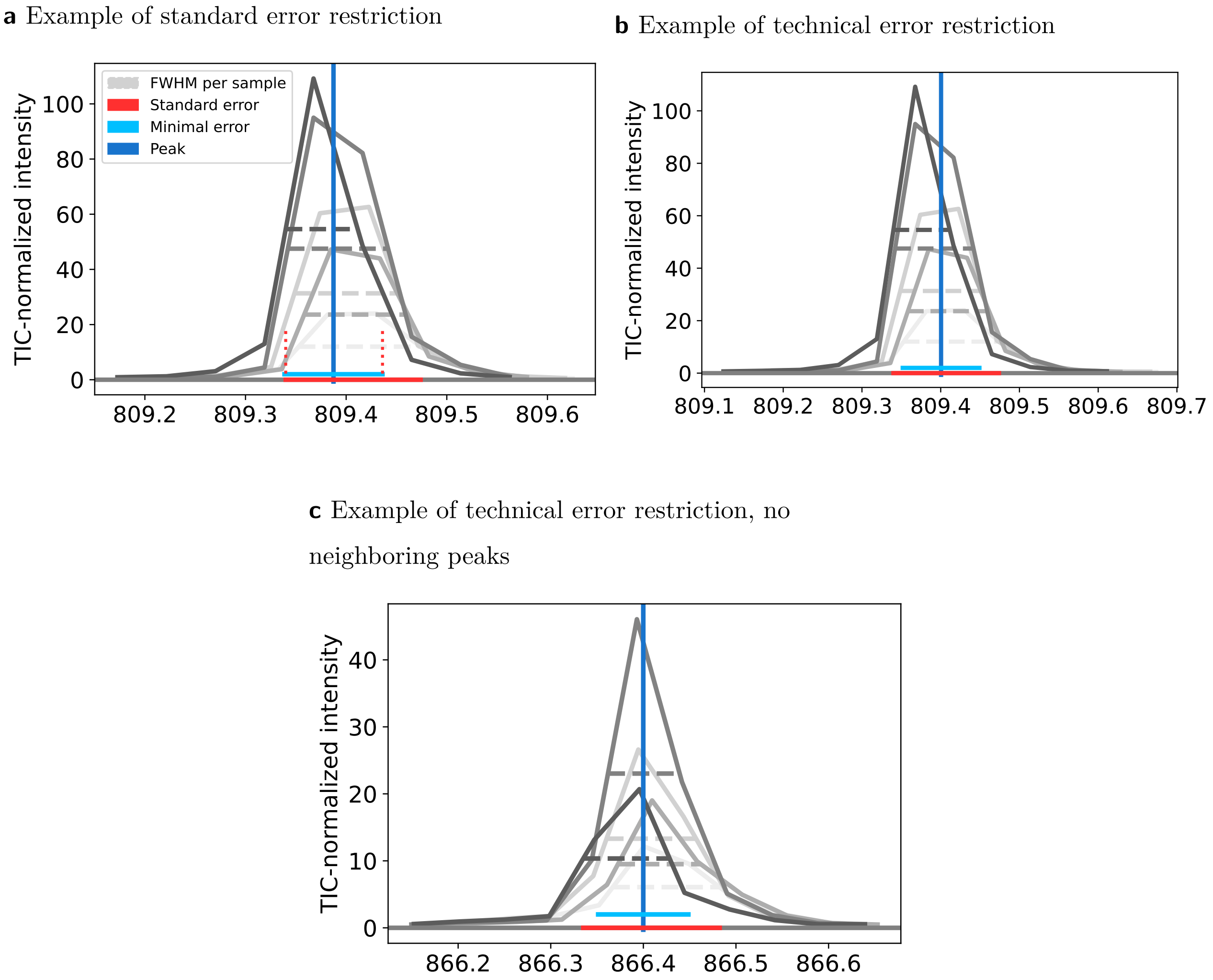

Supplementary Figure 17: Defining mass spectrometry imaging (MSI) mass ranges *MSI mass min* and *MSI mass max* of peaks for mapping with tandem mass spectrometry (MS) masses. **a** Standard error (red line), given by the full width at half maximum (FWHM) of all samples, restricts the minimum error (blue line) at peak - 0.0487. **b** Minimum error of peak is within the range of the standard error and is not further restricted. **c** Peak without (preserved) neighboring peaks that could be linked to mass shifts. Minimum error leads to a more conservative range used for mapping with tandem MS masses than the standard error.

Supplementary tables

Supplementary Table 1: Peptide candidates (#43) found with at least 2 masses matched from semi-supervised convolutional autoencoder (ConvAE) run to tandem mass spectrometry experiment, showing only one exemplary mass pair, the complete data is provided as Supplementary Table in a separate file

Protein(s)	Gene name(s)	Mass 1	Mass 2
Keratin, type II cytoskeletal 6A;Keratin, type ...	KRT6A;KRT6C;KRT6B	808.387	877.441
Cytochrome b-c1 complex subunit 1, mitochondrial	UQCRC1	808.400	1042.519
Phosphoglycerate kinase 1	PGK1	808.400	1011.519
Multifunctional protein ADE2;Phosphoribosylamin...	PAICS	1015.501	1409.733
Prelamin-A/C;Lamin-A/C	LMNA	1042.547	971.497
Trifunctional purine biosynthetic protein adeno...	GART	1042.547	1036.548
Heterogeneous nuclear ribonucleoprotein M	HNRNPM	1425.709	822.371
Keratin, type II cytoskeletal 5	KRT5	1425.709	1424.693
60S ribosomal protein L18a	RPL18A	1425.709	1042.519
60S ribosomal protein L15;Ribosomal protein L15	RPL15	1425.709	1166.612
Cullin-associated NEDD8-dissociated protein 1	CAND1	988.480	965.469
Serine hydroxymethyltransferase, mitochondrial;...	SHMT2	988.480	854.495
Heat shock cognate 71 kDa protein	HSPA8	988.480	1409.694
DNA-dependent protein kinase catalytic subunit	PRKDC	877.466	1337.665
Eukaryotic translation initiation factor 3 subu...	EIF3C;EIF3CL	1042.519	1166.637
Vigilin	HDLBP	1424.713	1061.512
Signal transducer and activator of transcriptio...	STAT1	1424.713	1307.631
Phosphoglucomutase-1	PGM1	1089.554	1026.520
V-type proton ATPase subunit B, brain isoform	ATP6V1B2	1089.554	1307.631
Keratin, type I cytoskeletal 14	KRT14	1424.693	1166.637
L-lactate dehydrogenase A chain	LDHA	1166.637	1026.520
Annexin A7	ANXA7	1043.548	1090.536
Hexokinase-1	HK1	1409.733	1030.522
Keratin, type I cytoskeletal 16	KRT16	1337.665	854.495
60S ribosomal protein L5	RPL5	1337.665	1000.473
Heterogeneous nuclear ribonucleoproteins A2/B1	HNRPA2B1;HNRNPA2B1	1337.665	1409.694
Collagen alpha-3(VI) chain	COL6A3	1036.529	989.471
Plastin-2	LCP1	997.502	1116.535
Programmed cell death protein 6	PDCD6	997.502	1338.656
Desmoplakin	DSP	1011.490	944.515

Supplementary Table 1: Continued: Peptide candidates (#43) found with at least 2 masses matched from semi-supervised convolutional autoencoder (ConvAE) run to tandem mass spectrometry experiment, showing only one exemplary mass pair, the complete data is provided as Supplementary Table in a separate file

Protein(s)	Gene name(s)	Mass 1	Mass 2
Gasdermin-A	GSDMA	944.493	1038.511
Basic leucine zipper and W2 domain-containing p...	BZW1	1061.512	807.391
Elongation factor 2	EEF2	1090.536	1038.511
Annexin A6	ANXA6	1090.536	1025.527
Protein-glutamine gamma-glutamyltransferase K	TGM1	971.497	1410.739
Eukaryotic translation initiation factor 3 subu...	EIF3L	989.471	964.486
Galectin-7	LGALS7	909.463	856.443
T-complex protein 1 subunit eta	CCT7	909.463	944.515
Eukaryotic translation initiation factor 4 gamma 1	EIF4G1	1026.520	1410.739
Coatomer subunit alpha;Xenin;Proxenin	COPA	807.391	944.515
Plectin	PLEC	1030.522	966.486
Glyceraldehyde-3-phosphate dehydrogenase	GAPDH	1410.739	1064.539
Importin-5	IPO5	1036.548	1116.535

Supplementary Table 2: Dependency of the dimensions of the latent representations (last column) on the input patch size (second column), illustrated using four exemplary input patch sizes

Largest image dimension (px.)	Patch size (x)	Padded image dimension (px.)	No. of patches per dimension	Latent space dimension (px.)
127	2	128	64	64
127	3	129	43	86
127	4	128	32	96
128	5	130	26	104
