# Supplementary material for: Enhancing mass spectrometry imaging accessibility using convolutional autoencoders for deriving hypoxia-associated peptides from tumors
Source: NPJ Syst Biol Appl. 2024 May 27;10:57. doi: 10.1038/s41540-024-00385-x (PMC11130291; doi:10.1038/s41540-024-00385-x)
Supplement: Supplementary file 2 — Reporting Summary [file 41540_2024_385_MOESM2_ESM.pdf]

nature portfolio

Corresponding author(s): Verena Bitto

Last updated by author(s): Apr 1, 2024

Reporting Summary

Nature Portfolio wishes to improve the reproducibility of the work that we publish. This form provides structure for consistency and transparency in reporting. For further information on Nature Portfolio policies, see our Editorial Policies and the Editorial Policy Checklist.

Statistics

For all statistical analyses, confirm that the following items are present in the figure legend, table legend, main text, or Methods section.

n/a	Confirmed	
☐	☒	The exact sample size (*n*) for each experimental group/condition, given as a discrete number and unit of measurement
☐	☒	A statement on whether measurements were taken from distinct samples or whether the same sample was measured repeatedly
☐	☒	The statistical test(s) used AND whether they are one- or two-sided<br>*Only common tests should be described solely by name; describe more complex techniques in the Methods section.*
☐	☒	A description of all covariates tested
☐	☒	A description of any assumptions or corrections, such as tests of normality and adjustment for multiple comparisons
☒	☐	A full description of the statistical parameters including central tendency (e.g. means) or other basic estimates (e.g. regression coefficient) AND variation (e.g. standard deviation) or associated estimates of uncertainty (e.g. confidence intervals)
☒	☐	For null hypothesis testing, the test statistic (e.g. *F*, *t*, *r*) with confidence intervals, effect sizes, degrees of freedom and *P* value noted<br>*Give P values as exact values whenever suitable.*
☒	☐	For Bayesian analysis, information on the choice of priors and Markov chain Monte Carlo settings
☒	☐	For hierarchical and complex designs, identification of the appropriate level for tests and full reporting of outcomes
☒	☐	Estimates of effect sizes (e.g. Cohen's *d*, Pearson's *r*), indicating how they were calculated

Our web collection on statistics for biologists contains articles on many of the points above.

Software and code

Policy information about availability of computer code

Data collection	For MSI data collection, flexControl (4.2) and FlexImaging (6.0) by Bruker was utilized.
Data analysis	For MSI pre-processing the R package Cardinal (2.10.0) was used. Image co-registration was performed by means of ITKElastix (0.17.1).<br>Data analysis for LC-MS/MS was carried out by MaxQuant (version 1.6.14.0).<br>Tensorflow (2.12.0) was utilized for implementing the convolutional autoencoder approaches using Python (3.8.8). The RF models were built using sklearn (1.3.0). Boxplots were created with statannotations (0.6.0) and seaborn (0.12.2), with statistical tests being performed in scipy (1.10.1).<br>Custom code is provided via GitHub: https://github.com/DKFZ-ABI/automsi

For manuscripts utilizing custom algorithms or software that are central to the research but not yet described in published literature, software must be made available to editors and reviewers. We strongly encourage code deposition in a community repository (e.g. GitHub). See the Nature Portfolio guidelines for submitting code & software for further information.

Data

Policy information about availability of data

All manuscripts must include a data availability statement. This statement should provide the following information, where applicable:

- Accession codes, unique identifiers, or web links for publicly available datasets
- A description of any restrictions on data availability
- For clinical datasets or third party data, please ensure that the statement adheres to our policy

The mass spectrometry proteomics data (both, MSI and LC-MS/MS) have been deposited to the ProteomeXchange Consortium via the PRIDE partner repository with the dataset identifier PXD047820.
https://www.ebi.ac.uk/pride/archive

Research involving human participants, their data, or biological material

Policy information about studies with human participants or human data. See also policy information about sex, gender (identity/presentation), and sexual orientation and race, ethnicity and racism.

Reporting on sex and gender	
Reporting on race, ethnicity, or other socially relevant groupings	*Please specify the socially constructed or socially relevant categorization variable(s) used in your manuscript and explain why they were used. Please note that such variables should not be used as proxies for other socially constructed/relevant variables (for example, race or ethnicity should not be used as a proxy for socioeconomic status).*<br>*Provide clear definitions of the relevant terms used, how they were provided (by the participants/respondents, the researchers, or third parties), and the method(s) used to classify people into the different categories (e.g. self-report, census or administrative data, social media data, etc.)*<br>*Please provide details about how you controlled for confounding variables in your analyses.*
Population characteristics	
Recruitment	*Describe how participants were recruited. Outline any potential self-selection bias or other biases that may be present and how these are likely to impact results.*
Ethics oversight	*Identify the organization(s) that approved the study protocol.*

Note that full information on the approval of the study protocol must also be provided in the manuscript.

Field-specific reporting

Please select the one below that is the best fit for your research. If you are not sure, read the appropriate sections before making your selection.

☒ Life sciences ☐ Behavioural & social sciences ☐ Ecological, evolutionary & environmental sciences

For a reference copy of the document with all sections, see nature.com/documents/nr-reporting-summary-flat.pdf

Life sciences study design

All studies must disclose on these points even when the disclosure is negative.

Sample size	For training the proposed autoencoder, overlapping patches from three untreated samples of xenograft tumor models (CAL33) were utilized. Additionally, overlapping patches from two further samples of the same tumor models were used to test the autoencoder. No overfitting on the training data was detected when inspecting the loss over time, suggesting that the model will generalize well to unseen data.
Data exclusions	The two samples used for testing the autoencoder, were not used for training the subsequent random forest models, given that the number of hypoxic regions were too few.
Replication	Autoencoder and random forest runs were conducted multiple times, with results described in the manuscript.
Randomization	Not applicable for this manuscript, however the xenograft models used were part of a larger project on the effect of hypoxia with details of the randomization process being described in Koi and Bitto et al. (https://doi.org/10.1186/s12967-023-04439-2)
Blinding	Not applicable for this manuscript, however the xenograft models used were part of a larger project on the effect of hypoxia with details of the randomization process being described in Koi and Bitto et al. (https://doi.org/10.1186/s12967-023-04439-2)

Reporting for specific materials, systems and methods

We require information from authors about some types of materials, experimental systems and methods used in many studies. Here, indicate whether each material, system or method listed is relevant to your study. If you are not sure if a list item applies to your research, read the appropriate section before selecting a response.

Materials & experimental systems

n/a	Involved in the study	
☐	☒	Antibodies
☐	☒	Eukaryotic cell lines
☒	☐	Palaeontology and archaeology
☐	☒	Animals and other organisms
☒	☐	Clinical data
☒	☐	Dual use research of concern
☒	☐	Plants

Methods

n/a	Involved in the study	
☒	☐	ChIP-seq
☒	☐	Flow cytometry
☒	☐	MRI-based neuroimaging

Antibodies

Antibodies used	Anti-pimonidazole polyclonal antibody (PAb2627, Hypoxyprobe, Inc (HPI), Burlington, USA) followed by incubation with FITC-conjugated anti rabbit secondary antibody to visualize hypoxic regions were used.
Validation	The protocol was established in our lab by Yaronoma et al. 2006 (doi: 10.1016/j.radonc.2006.08.010) and is used since then. Isotype control stainings using the secondary antibody were performed.

Eukaryotic cell lines

Policy information about cell lines and Sex and Gender in Research

Cell line source(s)	Cal33 was bought from the Leibniz Institute DSMZ-German Collection of Microorganisms and Cell Cultures,
Authentication	Cal33 was authentified via STR profiling.
Mycoplasma contamination	Our cell lines are regularily tested for mycoplasma contamination.
Commonly misidentified lines (See ICLAC register)	-

Animals and other research organisms

Policy information about studies involving animals; ARRIVE guidelines recommended for reporting animal research, and Sex and Gender in Research

Laboratory animals	7–14 week-old male and female NMRI (nu/nu)
Wild animals	-
Reporting on sex	Male and female animals were used for tumor generation
Field-collected samples	-
Ethics oversight	The animal facility and the experiments followed the ARRIVE guidelines and were approved according to the institutional guidelines and the German animal welfare regulations

Note that full information on the approval of the study protocol must also be provided in the manuscript.

Plants

Seed stocks	*Report on the source of all seed stocks or other plant material used. If applicable, state the seed stock centre and catalogue number. If plant specimens were collected from the field, describe the collection location, date and sampling procedures.*
Novel plant genotypes	*Describe the methods by which all novel plant genotypes were produced. This includes those generated by transgenic approaches, gene editing, chemical/radiation-based mutagenesis and hybridization. For transgenic lines, describe the transformation method, the number of independent lines analyzed and the generation upon which experiments were performed. For gene-edited lines, describe the editor used, the endogenous sequence targeted for editing, the targeting guide RNA sequence (if applicable) and how the editor was applied.*
Authentication	*Describe any authentication procedures for each seed stock used or novel genotype generated. Describe any experiments used to assess the effect of a mutation and, where applicable, how potential secondary effects (e.g. second site T-DNA insertions, mosiacism, off-target gene editing) were examined.*
